# RNA Polymerase RPOTp is Involved in C‐to‐U RNA Editing at Multiple Sites in *Arabidopsis* Chloroplasts

**DOI:** 10.1002/advs.202405131

**Published:** 2024-12-04

**Authors:** Nadia Ahmed Ali, Wenjian Song, Yayi Zhang, Jiani Xing, Kexing Su, Xingxing Sun, Yujia Sun, Yizhou Jiang, Dianxing Wu, Xiaobo Zhao

**Affiliations:** ^1^ Key Laboratory of Nuclear Agricultural Sciences of Ministry of Agriculture and Rural Affairs Key Laboratory of Nuclear Agricultural Sciences of Zhejiang Province Institute of Nuclear Agricultural Sciences College of Agriculture and Biotechnology Zhejiang University Hangzhou 310058 China

**Keywords:** chloroplast, MORF, RNA editing, RNA polymerase, RPOTp

## Abstract

RPOTp is the nuclear‐encoded plastid‐targeted RNA polymerase and plays a crucial role in chloroplast gene expression. Transcripts in plant organelles are altered by the conversion of cytidine (C) to uridine (U) at specific positions through RNA editing. However, whether RPOTp is involved in chloroplast RNA editing remains unclear. Here, the role of RPOTp in C‐to‐U RNA editing at multiple sites in *Arabidopsis* chloroplasts is uncovered. Multiple organellar RNA editing factor 2 (MORF2) is required for the editing of most sites in chloroplasts. RPOTp is identified from the co‐immunoprecipitation targets of MORF2. The *sca3‐2* mutant, defective in RPOTp, exhibits a pale‐yellow phenotype and alters the RNA editing of nine sites in chloroplasts. It is also shown that RNA editing is uncoupled from chloroplast transcriptional activity. RPOTp directly interacts with chloroplast multiple‐site RNA editing factors, including MORF2, MORF8, MORF9, and ORRM1. It is further shown that RPOTp participates in RNA editing by influencing the dimerization of MORF proteins. The defect in RPOTp impairs the expression of most chloroplast genes, indicating an indispensable role for RPOTp in chloroplast gene expression. These findings reveal that RPOTp not only participates in transcription but also has a novel role in RNA editing of chloroplast transcripts.

## Graphical abstract

This study shows that RPOTp, the nucleus‐encoded RNA polymerase in the chloroplast transcription machinery, participates in multiple‐site RNA editing in the chloroplast. RPOTp directly interacts with MORF2, MORF8, MORF9, and ORRM1, affecting the dimerization of MORF proteins in chloroplast RNA editosomes. The dual role of RPOTp in chloroplast gene transcription and RNA editing is essential for chloroplast gene expression and development.

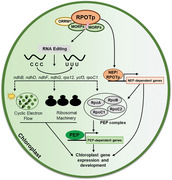

## 1 Introduction

Chloroplasts are essential organelles for plant growth and development due to their crucial role in photosynthesis, and they also serve as a site for the production of many hormones and metabolites.^[^ ^1^ ^]^ The biogenesis of chloroplasts is a highly complex process that requires the expression of a specific set of both chloroplast‐encoded and nuclear‐encoded genes.^[^ ^2^ ^]^ Despite the small number of genes encoded by the chloroplast genome, the transcription and transcript processing of higher‐plant chloroplast genes is extremely complex. Chloroplast genes are transcribed by a plastid‐encoded RNA polymerase (PEP) and one (monocots) or two (dicots) nucleus‐encoded RNA polymerase(s) (NEP).^[^ ^3^ ^]^ PEP is a bacterial‐type multisubunit enzyme composed of core subunits encoded by plastidial *rpoA*, *rpoB*, *rpoC1*, and *rpoC2* genes and nucleus‐encoded sigma factors. Six sigma factors are required by PEP for promoter recognition in *Arabidopsis* plastids.^[^ ^3^ ^]^ In contrast, NEP is composed of a single catalytic subunit that performs the transcription process and transcribes the PEP core subunits and components of the plastid genetic system.^[^ ^3^ ^]^ NEP is encoded by the *RPOT* (RNA polymerase of the phage T3/T7 type) genes that are represented as RPOTp (chloroplast‐targeted RPOT), RPOTm (mitochondrion‐targeted RPOT), and RPOTmp (chloroplast and mitochondrion dual‐targeted RPOT) in *Arabidopsis*.^[^ ^4^ ^]^ RPOTp is exclusively localized to plastids, whereas RPOTmp participates in the transcription of plastid and mitochondrial genes.^[^ ^5^ ^]^ The RPOTp coding gene is called *SCABRA3* (*SCA3*). The knockout line of *SCA3* exhibited stunted growth with severely impaired chloroplast development and altered leaf morphology.^[^ ^6^ ^]^ The transcripts of plastids usually undergo a complicated posttranscriptional modification process with the involvement of many nuclear‐encoded factors. These modifications include RNA editing, intron splicing, maturation of transcript ends, and RNA stabilization.^[^ ^7^ ^]^

RNA editing is a posttranscriptional modification of RNA that changes the identity of nucleotides in RNAs in the form of deletion, addition, or substitution so that the information in the mature RNA differs from that defined in the genome.^[^ ^8^ ^]^ In plants, RNA editing has been found for over 30 years.^[^ ^9^ ^]^ The conversion of cytidine (C) to uridine (U) in chloroplast and mitochondrial RNAs is the main type of RNA editing in land plants. There are ≈20–60 RNA editing sites in the chloroplast and over 600 sites in the mitochondrion of land plants.^[^ ^8^, ^10^, ^11^ ^]^ Recent studies have identified a number of nucleus‐encoded factors that constitute the editosome complex required for organellar RNA editing, such as pentatricopeptide repeat (PPR) proteins,^[^ ^12^ ^]^ multiple organelle RNA editing factors (MORFs, also known as RNA editing factor interacting proteins, RIPs),^[^ ^13^, ^14^ ^]^ organelle RNA recognition motif (ORRM) containing proteins,^[^ ^15^ ^]^ protoporphyrinogen IX oxidase 1 (PPO1)^[^ ^16^ ^]^ and organelle zinc finger 1 (OZ1).^[^ ^17^ ^]^

PPR proteins are the most common nucleus‐encoded factors in plant RNA editosomes. PPR proteins are classified into P and PLS subfamilies according to their domain compositions.^[^ ^18^, ^19^ ^]^ PLS‐subfamily PPR proteins mainly work as site‐recognition factors that bind to RNAs directly. Some PPRs can only target one editing site, while some can target multiple sites.^[^ ^7^, ^20^ ^]^ MORF proteins are also important components in plant RNA editosomes. The *Arabidopsis* MORF family has nine members, among which MORF2 and MORF9 control the editing of almost all sites in chloroplasts.^[^ ^13^, ^14^ ^]^ MORF proteins can form homomers or heteromers and also selectively interact with other RNA editing factors such as PPRs.^[^ ^13^, ^14^, ^20^ ^]^ The association between MORF9 and PPR can increase the affinity of the PPR motifs to target RNAs.^[^ ^21^, ^22^ ^]^ In addition, MORF proteins also bring together PPR‐E/E+ and PPR‐DYW proteins, which provides the specificity for editing sites and cytidine deaminases.^[^ ^9^, ^23^, ^24^, ^25^ ^]^ MORF families have also been identified in other plant species, including *Populus trichocarpa* with nine members,^[^ ^26^ ^]^ rice with seven,^[^ ^27^ ^]^ *Zea mays* with seven,^[^ ^28^ ^]^ *Actinidia chinensis* with ten^[^ ^29^ ^]^ and *Nicotiana tabacum* with eight members.^[^ ^30^ ^]^ These MORF proteins also play an essential role not only in RNA editing but also in responding to various stress conditions, as seen in poplar, where MORF proteins respond to drought stress, and in *Nicotiana tabacum*, where NbMORF8 was found to negatively regulate the plant immunity to pathogens.^[^ ^26^, ^30^ ^]^

Although the mutant phenotype of *SCA3* gene has been reported before,^[^ ^6^ ^]^ the role of RPOTp in chloroplast RNA editing has not been investigated. In this study, we find that RPOTp is involved in the C‐to‐U RNA editing of multiple sites as well as the correct processing of various transcripts in chloroplasts, thereby controlling the activity of PEP and NEP to regulate the expression of chloroplast genes. In addition, RPOTp interacts with known chloroplast multiple‐site RNA editing factors, including MORF2, MORF8, MORF9, and ORRM1. We further show that RPOTp participates in RNA editing by affecting the dimerization of MORF proteins in chloroplast editosomes. Based on these results, we propose that RNA polymerase RPOTp has a novel role in affecting the editing efficiency of chloroplast transcripts in *Arabidopsis*.

## 2 Results

### 2.1 RPOTp Interacts with RNA Editing Factor MORF2 in Chloroplasts

**Figure 1:**
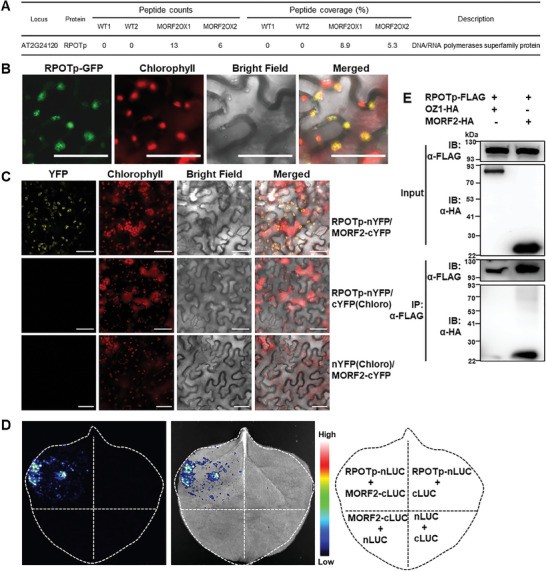
RPOTp interacts with MORF2 in chloroplasts. A) Peptide counts and coverage for RPOTp identified by Co‐IP of MORF2 followed by mass spectrometric analysis.^[^ ^31^ ^]^ WT: wild‐type negative control. MORF2OX: MORF2 overexpression line. B) The subcellular localization of RPOTp in *Nicotiana benthamiana* epidermal cells. The red autofluorescence of chlorophyll was used as the indicator of chloroplasts. Bright‐field images correspond to epidermal cells. Merged images show the colocalization of GFP with chloroplasts. Scale bar, 50 µm. C) The interaction between RPOTp and MORF2 as shown by BiFC assay. Co‐infiltration of RPOTp‐nYFP and MORF2‐cYFP reconstitutes YFP signal, whereas no signal is detected when RPOTp‐nYFP/cYFP(Chloro) or MORF2‐cYFP/nYFP(Chloro) are co‐expressed. nYFP: N‐terminal YFP; cYFP: C‐terminal YFP. nYFP(Chloro) and cYFP(Chloro) represent chloroplast‐targeted nYFP and cYFP, respectively. The red autofluorescence of chlorophyll indicates the localization of chloroplasts. Bright‐field images correspond to the epidermal cells. Merged images show the colocalization of YFP with chloroplasts. Scale bar: 50 µm. D) Interaction between RPOTp and MORF2 in LCI assay. Co‐transformation of RPOTp‐nLUC and MORF2‐cLUC complemented the luciferase activity. nLUC: N‐terminal luciferase, cLUC: C‐terminal luciferase. Target proteins co‐transformed with empty plasmids were used as negative controls. E) RPOTp interacts with MORF2 in Co‐IP assay. RPOTp‐FLAG and MORF2‐HA, or OZ1‐HA were co‐infiltrated into *Nicotiana benthamiana* leaves and FLAG beads were used for immunoprecipitation. Gel blots were probed with anti‐FLAG or anti‐HA antibody. IB: immunoblotting; IP: immunoprecipitation.

Previously, we carried out a coimmunoprecipitation (Co‐IP) assay followed by mass spectrometric analysis for the important plastid RNA editosome component MORF2 to identify MORF2‐binding proteins.^[^ ^31^ ^]^ The RPOTp protein encoded by *AT2G24120* (*SCABRA3: SCA3*) was selected for further investigation as it had abundant matches in the mass spectrometric analysis and was not detected in negative controls (**Figure** **1**A). Although RPOTp is reported as the plastid‐localized RNA polymerase, no fluorescence protein‐tagged subcellular localization has been examined for it. Thus, we confirmed the chloroplast localization of RPOTp by transiently expressing the RPOTp‐GFP (green fluorescence protein) fusion in *Nicotiana benthamiana* epidermal cells. Strong GFP signals in dots were detected, and these GFP signals co‐localized with red autofluorescence signals of chlorophyll (Figure 1B), indicating the chloroplast localization of RPOTp.

To validate the direct interaction between RPOTp and MORF2, we performed three protein‐protein interaction assays. For bimolecular fluorescence complementation (BiFC) assay, co‐expression of RPOTp‐nYFP (YFP N‐terminal fragment fusion) and MORF2‐cYFP (YFP C‐terminal fragment fusion) reconstituted YFP fluorescence in chloroplasts, demonstrating that RPOTp directly interacts with MORF2. As negative controls, RPOTp‐cYFP or MORF2‐nYFP were co‐expressed with chloroplast‐localized nYFP or cYFP (by the transit peptide of chloroplast‐localized OTP81),^[^ ^31^ ^]^ respectively, and no YFP signals were observed (Figure 1C). The firefly luciferase complementation imaging (LCI) assay also showed the interaction of RPOTp with MORF2. Co‐expression of RPOTp‐nLUC (luciferase N‐terminal fragment fusion) and MORF2‐cLUC (luciferase C‐terminal fragment fusion) led to high levels of luciferase activity, whereas negative controls showed no luciferase activity (Figure 1D). The interaction between RPOTp and MORF2 was further confirmed by Co‐IP. FLAG‐tagged RPOTp, HA‐tagged MORF2, or HA‐tagged OZ1 (negative control) were co‐infiltrated into *Nicotiana benthamiana* leaves. MORF2 protein was co‐immunoprecipitated with RPOTp, while OZ1 proteins were not, indicating that RPOTp interacts with MORF2 (Figure 1E). Together, these results demonstrate that RPOTp can directly interact with MORF2.

### 2.2 Phenotypic Characterization and Complementation of the *sca3‐2* Mutant

To characterize the function of RPOTp in chloroplast RNA editing, we obtained the T‐DNA insertional mutant named *sca3‐2* (SALK_093884) for the *SCA3* gene as the study object. The *sca3‐2* mutant contains a T‐DNA insertion in the third intron of *SCA3* gene (Figure S1A, Supporting Information). The T‐DNA insertion in *SCA3* was confirmed by the amplification of a specific band in the homozygous mutant plants (Figure S1B, Supporting Information). The *sca3‐2* mutant plants display smaller pale‐yellow cotyledons and seedling leaves, as well as smaller plants throughout their entire life cycle compared to Col‐0 wild type when grown under standard light conditions (Figures S1C and S2A, Supporting Information). qRT‐PCR analysis shows that the expression level of *SCA3* decreases to ≈5% of the wild type level in 4‐day‐old *sca3‐2* mutant plants (Figure S2B, Supporting Information), while reduced to ≈15% in 10‐day‐old *sca3‐2* mutant plants (Figure S2C, Supporting Information). We also examined the ultrastructure of chloroplasts, the results showed that chloroplasts in *sca3‐2* mutant dramatically reduced in number and not properly developed. Chloroplasts in the *sca3‐2* mutant were small, and their shape was abnormal. The thylakoids were severely disrupted in the *sca3‐2* mutant, and large thylakoid lamellas and transparent vacuoles were also observed in some chloroplasts. Some plastids formed rudimentary thylakoids consisting only of grana lamellae and failed to accumulate stromal lamellae, while others were filled with numerous vesicles (Figure S3, Supporting Information). We generated complementary plants (*sca3‐2/com*) of *sca3‐2* mutants. A representative complementary T3 plant (*sca3‐2/com1*) is shown in Figures S1C and S2A (Supporting Information), displaying normal green cotyledons and leaves phenotype. The complementary plants also restored the seedling growth and development as of the wild type Col‐0 (Figures S1C and S2A, Supporting Information). Chlorophyll is one of the most important indicators of leaf color change, we also examined the chlorophyll contents in the Col‐0, *sca3‐2*, and *sca3‐2/com1* seedlings. The chlorophyll content in *sca3‐2* mutants is significantly lower compared with Col‐0, and in *sca3‐2/com1* plants, it restores to the level of the wild type (Figure S1D, Supporting Information). In complementary plants, although *SCA3* is driven by its native promoter, the expression level of *SCA3* gene is ≈3 times that of the wild type (Figure S2B,C, Supporting Information), which is common in transgenic plants.

### 2.3 The RPOTp Loss of Function Affects RNA Editing Efficiency at Multiple Sites in Chloroplasts

**Figure 2:**
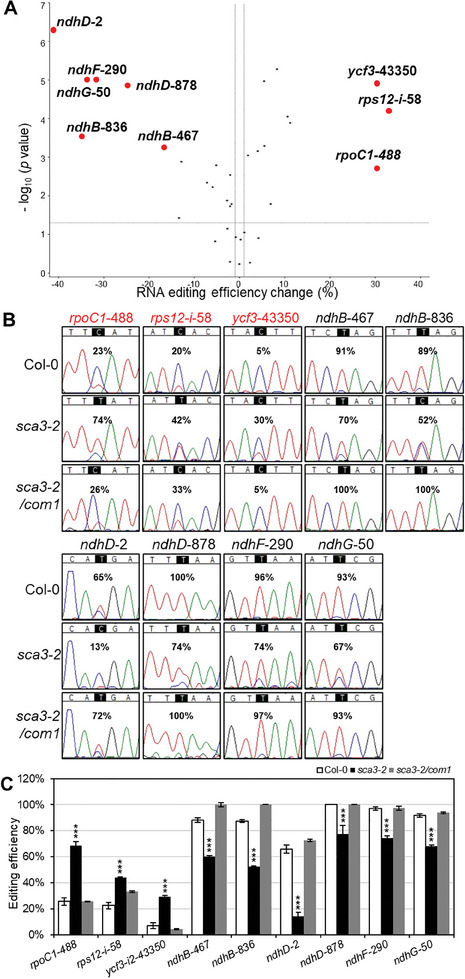
Chloroplast RNA editing defects in the *sca3‐2* mutant. A) Volcano plot of RNA editing efficiency change in the *sca3‐2* mutant compared to the Col‐0. On the *y*‐axis, the negative log10 *p* values are plotted. On the *x*‐axis, RNA editing efficiency changes (%) are shown in the RNA‐seq analysis. Red dots represent the editing sites with significant changes (efficiency change > 15%; *p* < 0.001). B) Sequencing chromatogram show the RNA editing efficiency (%) of sites affected in the *sca3‐2* mutant compared with the Col‐0 wild type. The edited sites are highlighted by dark blocks, and the calculated C to T (equal C to U in RNA) editing efficiencies are labeled. The affected sites names are labeled at the top, and the names of sites with increased editing efficiency are labeled in red. The peak for C is in blue, and the peak for T is in red. C) Chloroplast RNA editing profiles of *sca3* mutation‐affected sites in Col‐0*, sca3‐2*, and *sca3‐2/com1* seedlings. The *x*‐axis indicates RNA editing sites. The *y*‐axis represents the editing efficiency. Data are the mean ± SEM from three biological replicates. Asterisks represent the significance level (^***^ *p* < 0.001) compared with Col‐0 using a two‐tailed Student's *t*‐test.

Considering the interaction between RPOTp and MORF2, we investigated the potential role of RPOTp in RNA editing. We used the RNA samples from 4‐day‐old plants as *SCA3* had a relatively low expression level in the *sca3‐2* mutant at the 4‐day‐old stage (Figure S2B, Supporting Information). Thus, we analyzed the RNA editing efficiency in the 4‐day‐old seedlings of *sca3‐2* and Col‐0 wild type using rRNA‐depleted RNA sequencing followed by Chloroseq analysis pipeline. The results showed that the *sca3‐2* mutant exhibited a significant difference in chloroplast RNA editing efficiency compared with the Col‐0 wild type. The editing efficiency of nine sites was significantly affected (efficiency change > 15%; *p* < 0.001) in the *sca3‐2* mutant (**Figure** **2**A and Table S1, Supporting Information). We also analyzed the chloroplast RNA editing efficiency between Col‐0 wild type and *sca3‐2* mutant using RT‐PCR and Sanger sequencing. The results showed that the editing efficiencies of above mentioned nine sites have either decrease or increase more than 20% in the *sca3‐2* mutant compared with the wild type. The editing efficiency of the *rpoC1*‐488 site markedly increased from 23% in Col‐0 to 74% in the *sca3‐2* mutant. Similarly, *rps12‐i*‐58 and *ycf3*‐43350 sites also showed increased editing efficiency in the *sca3‐2* mutant, while *rps12‐i*‐58 increasing from 20% to 42% and *ycf3*‐43350 increasing from 5% to 30% compared to Col‐0 wild type (Figure 2B,C). On the contrary, the editing efficiency for *ndhB*‐467 in the Col‐0 wild type is ≈91%, whereas in *sca3‐2*, it is reduced to ≈70%. The editing efficiency of the *ndhB*‐836 site also decreased from 89% in Col‐0 to 52% in the *sca3‐2* mutant plants. The disruption of *SCA3* severely affects the editing of *ndhD*‐2 site, which decreases by 52%. The *ndhD*‐878 site is 100% edited in Col‐0 but has a reduced editing efficiency of 74% in the *sca3‐2* mutant. *ndhF*‐290 editing dropped from 96% in the wild type to 74% in the mutant, while the editing of *ndhG*‐50 dropped from 93% to 67% (Figure 2B,C). The editing efficiencies of the remaining chloroplast RNA editing sites are not significantly affected or have changes of less than 20% in the *sca3‐2* mutant revealed by Sanger sequencing (Figure S4, Supporting Information). We further analyzed RNA‐editing efficiency differences in 10‐day‐old seedlings of the *sca3‐2* mutant and Col‐0 wild type. The result showed that RNA editing efficiencies were also increased for *rpoC1*‐488 and *rps12*‐*i*‐58 sites and decreased for *ndhB*‐836, *ndhB*‐467, *ndhD*‐2, *ndhD*‐878, *ndhF*‐290, and *ndhG*‐50 compared with Col‐0 wild type (Figure S5, Supporting Information). RNA editing analysis was also performed on a complementary line of the *sca3‐2* mutant. The results show that complementation is not only observed in plant phenotypes but also in RNA editing efficiency in the *sca3‐2/com1* transgenic line (Figure 2B,C).

### 2.4 The Transcription Level and RNA Editing Efficiency of Chloroplast Genes are Uncoupled

**Figure 3:**
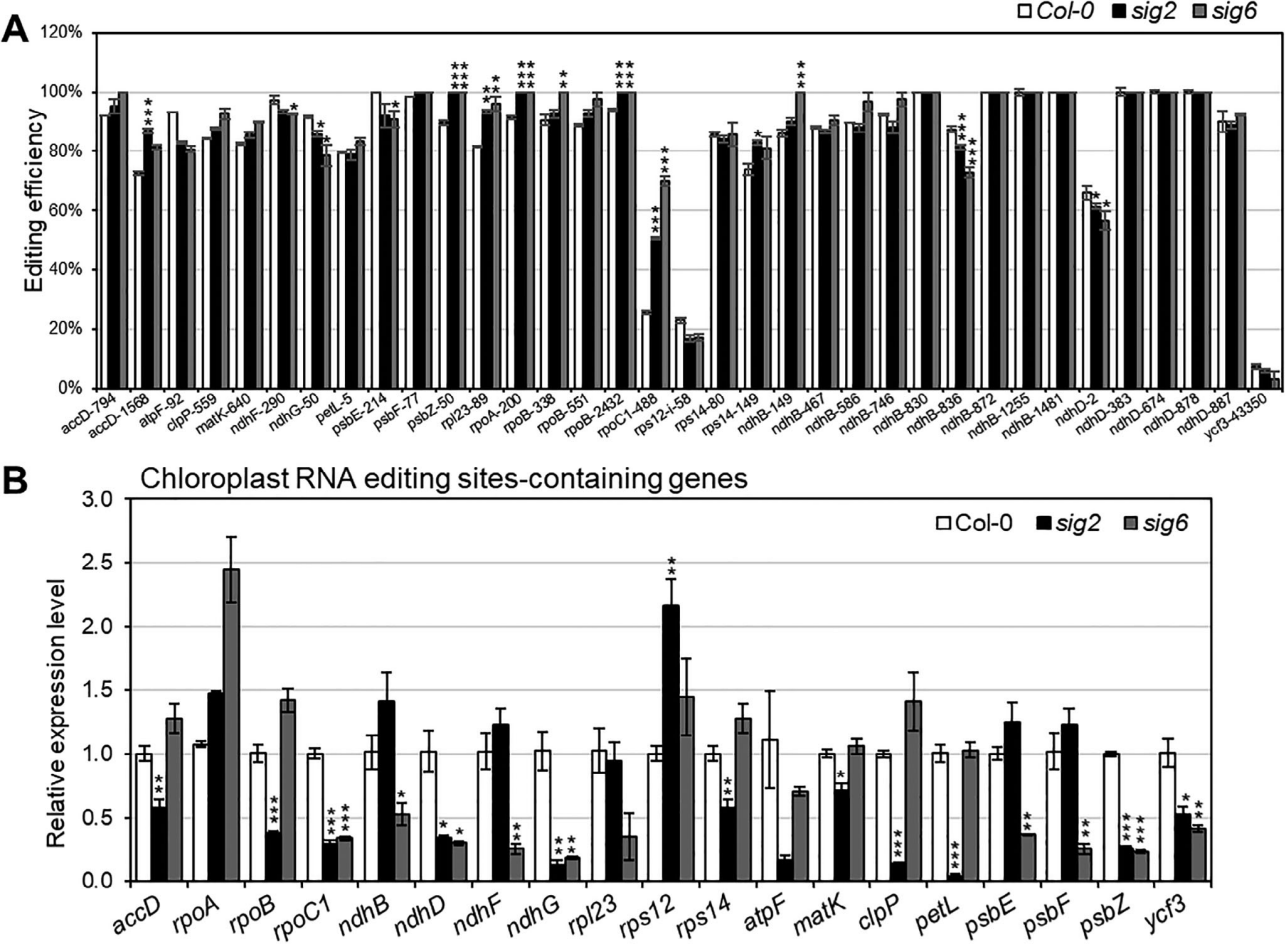
RNA editing profiles of chloroplast sites in 4‐day‐old seedlings of *sig2*, *sig6*, and Col‐0 wild type. A) The *x*‐axis indicates different RNA editing sites. The *y*‐axis represents the editing efficiency of each site. Data are the mean ± SEM from three biological replicates. Asterisks represent significance levels of ^*^ *p* < 0.05, ^**^ *p* < 0.01, ^***^ *p* < 0.001 (two‐tailed Student's *t*‐test) compared with Col‐0 wild type. B) Relative expression levels of chloroplast RNA editing sites‐containing genes in *sig2* and *sig6* mutants as compared to Col‐0 wild type in 4‐day‐old plants. Values are calculated from three biological replicates by normalizing against *PP2AA3*. Data are the mean ± SEM from three biological replicates, and asterisks indicate a statistical difference compared with Col‐0 wild type (^*^ *p* < 0.05, ^**^ *p* < 0.01, ^***^ *p* < 0.001) using a two‐tailed Student's *t*‐test.

Previous studies reported that mutants of several RNA editing factors showed a pale or yellow phenotype with severely affected chloroplast development but only disrupted the editing of one or two sites. CLB19 is a PPR protein that is involved in chloroplast RNA editing. *clb19* mutants have a yellow phenotype with severely impaired chloroplast development and early seedling lethality, however, only the editing of *rpoA*‐200 and *clpP*‐559 is abolished in *clb19* mutants.^[^ ^32^ ^]^ Yellow seedling 1 (YS1) is also essential for chloroplast development during early growth, with a virescent phenotype in the mutant plant, and is only required for the editing of *rpoB*‐338.^[^ ^33^ ^]^ OsPPR6 is another PPR protein in rice, the absence of which showed early chloroplast developmental defects leading to albino leaves and seedling death, and only controls the editing of the *ndhB*‐737 site in chloroplasts.^[^ ^34^ ^]^ These previous studies have suggested that there is no correlation between RNA editing defects and albinism or the yellowish phenotype. Furthermore, we also examined whether defects in the transcription of chloroplast genes may affect RNA editing efficiencies of corresponding sites. We assayed the RNA editing profile in mutants of Sigma factor 2 (SIG2) and SIG6, which are components of the PEP complex and essential for the transcription of chloroplast genes. The results showed that pale yellow 4‐day‐old seedlings of *sig2* and *sig6* mutants^[^ ^35^ ^]^ showed no significant efficiency change (>20%) in chloroplast RNA editing sites, except for the *rpoC1*‐488 site (**Figure** **3**A). Additionally, the RNA editing sites of *psbE*‐214, *psbF*‐77, *psbZ*‐50, *ndhB*‐149, *ndhB*‐467, *ndhB*‐586, *ndhB*‐746, *ndhB*‐830, *ndhB*‐836, *ndhB*‐872, *ndhB*‐1255, *ndhB*‐1481, *ndhD*‐2, *ndhD*‐383, *ndhD*‐674, *ndhD*‐878, *ndhD*‐887, *ndhF*‐290, *ndhG*‐50, and *ycf3*‐43350 did not exhibit any significant RNA editing defects (>20% changes) in the *sig6* mutant (Figure 3A), despite the fact that the expression levels of *psbE*, *psbF*, *psbZ*, *ndhB*, *ndhD*, *ndhF*, *ndhG*, and *ycf3* transcripts are dramatically reduced in the *sig6* mutant (Figure 3B). Similarly, the transcript levels of *accD*, *rpoB*, *ndh* *D*, *ndhG*, *rps14*, *matK*, *clpP*, *psbZ*, *petL*, and *ycf3* genes are significantly decreased in the *sig2* mutant (Figure 3A), but the editing efficiency at RNA editing sites of these genes remained unaffected (Figure 3B). PAP2/pTAC2 and PAP7/pTAC14 are PEP‐associated proteins (PAP) also required for the transcription activity of the PEP complex. We further obtained the *Arabidopsis pap2* and *pap7* mutants (Figure S6A–C, Supporting Information) and examined their chloroplast RNA editing profiles. No significant RNA editing efficiency changes (>20%) were observed in 4‐day‐old *pap2* and *pap7* mutant plants (Figure S6D, Supporting Information), even though *pap2* and *pap7* mutants significantly changed the expression levels of most chloroplast RNA editing sites‐containing genes (Figure S6E, Supporting Information). All these results indicate that the transcription level and RNA editing efficiency of most RNA editing sites containing chloroplast genes are uncoupled. Thus, RNA editing defects in the *sca3‐2* mutant are unlikely to be a secondary effect caused by changes in the transcript itself or early chloroplast developmental defects, because editing defects are only observed in nine but not in all chloroplast RNA editing sites. Moreover, the editing events in the same transcript are not all affected in the same pattern by the *sca3‐2* mutation. For example, *ndhB*‐467 and *ndhB*‐836 sites of *ndhB* and *ndhD*‐2 and *ndhD*‐878 sites of *ndhD* transcripts have significantly decreased editing in the *sca3‐2* mutant, but remaining editing sites in *ndhB* (*ndhB*‐149, *ndhB*‐586, *ndhB*‐746, *ndhB*‐830, *ndhB*‐872, *ndhB*‐1255, *ndhB*‐1481) and *ndhD* (*ndhD*‐383, *ndhD*‐674, *ndhD*‐887) are not significantly affected (Figure S4, Supporting Information). All these results indicate that RPOTp plays a direct role in modulating the RNA editing efficiency of specific sites in *Arabidopsis* chloroplast transcripts.

### 2.5 RPOTp also Interacts with Known Chloroplast Multiple‐Site RNA Editing Factors MORF8, MORF9, and ORRM1

**Figure 4:**
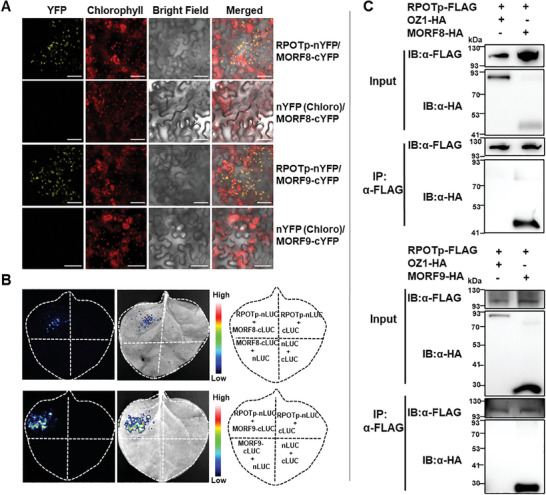
RPOTp interacts with MORF8 and MORF9. A) Detection of RPOTp interaction with MORF8/MORF9 by BiFC assay. Co‐expression of RPOTp‐nYFP and MORF8/MORF9‐cYFP leads to the reconstitution of YFP, whereas no signal is detected when RPOTp‐nYFP/cYFP(Chloro) or MORF8/MORF9‐cYFP/nYFP(Chloro) are co‐expressed. nYFP(Chloro) and cYFP(Chloro) represent chloroplast‐targeted nYFP and cYFP, respectively. The red autofluorescence of chlorophyll indicates the localization of chloroplasts. Bright‐field images correspond to the epidermal cells. Merged images show the colocalization of YFP with chloroplasts. Scale bar is 50 µm. B) Identification of interaction between RPOTp and MORF8/MORF9 by LCI assay. Co‐transformation of plasmids complemented the luciferase leading to high luciferase activity indicating the interaction. Target proteins co‐transformed with empty plasmids were used as negative controls. C) Interaction detection of RPOTp with MORF8/MORF9 in Co‐IP assay. RPOTp‐FLAG and MORF8/MORF9‐HA, or OZ1‐HA were co‐infiltrated into *Nicotiana benthamiana* leaves. FLAG beads were used to immunoprecipitate MORF8/MORF9‐HA and OZ1‐HA proteins. OZ1‐HA is the negative control. Gel blots were probed with the anti‐FLAG or anti‐HA antibody.

As RPOTp shows a direct interaction with MORF2, we further checked the possible interaction of RPOTp with the two‐remaining chloroplast‐localized MORF proteins (MORF8 and MORF9). For BiFC, co‐expression of RPOTp fused to nYFP and MORF8 or MORF9 fused to cYFP reconstituted YFP fluorescence in chloroplasts of *Nicotiana benthamiana* epidermal cells, while no YFP signals were detected in negative controls (**Figure** **4**A). Next, the LCI assay also confirmed the interaction between RPOTp and MORF8 or MORF9 (Figure 4B). We further constructed RPOTp‐FLAG, MORF8‐HA, and MORF9‐HA epitope tag vectors and performed the coimmunoprecipitation analysis. The FLAG antibodies were able to precipitate the MORF8‐HA and MORF9‐HA fusion proteins (Figure 4C).

**Figure 5:**
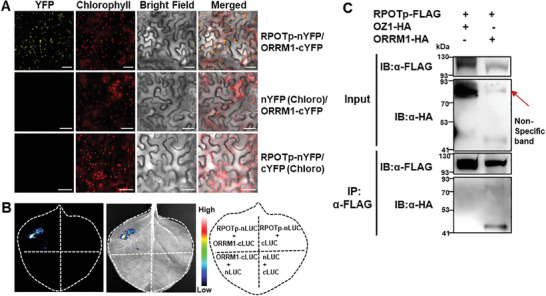
RPOTp interacts with ORRM1. A) Detection of the interaction between RPOTp and ORRM1 in tobacco leaf epidermal cells in BiFC assay. Co‐expression of RPOTp‐nYFP and ORRM1‐cYFP leads to the reconstitution of YFP, whereas no signal is detected when RPOTp‐nYFP or ORRM1‐cYFP are co‐expressed with cYFP(Chloro) or /nYFP(Chloro). nYFP(Chloro) and cYFP(Chloro) represent chloroplast‐targeted nYFP and cYFP, respectively and used as negative controls. The red autofluorescence of chlorophyll indicates the localization of chloroplasts. Bright‐field images correspond to the epidermal cells. Merged images show the colocalization of YFP with chloroplasts. Scale bar is 50 µm. B) The co‐transformation of RPOTp‐nLUC and ORRM1‐cLUC complements the luciferase activity in LCI assay. Target proteins co‐transformed with empty plasmids were used as negative controls. C) RPOTp interacts with ORRM1 in Co‐IP assay. RPOTp‐FLAG and ORRM1‐HA, or OZ1‐HA were co‐infiltrated into *Nicotiana benthamiana* leaves. FLAG beads were used to immunoprecipitate ORRM1‐HA and OZ1‐HA proteins. OZ1‐HA is the negative control. Gel blots were probed with anti‐FLAG or anti‐HA antibody.

ORRM1 is another important chloroplast multiple‐site RNA editosome component and the mutation of ORRM1 impairs chloroplast RNA editing at 12 sites in *Arabidopsis*.^[^ ^15^ ^]^ We also detected the interaction of RPOTp with ORRM1 in BiFC and LCI assays (**Figure** **5**A,B). The Co‐IP assay further confirmed the interaction between RPOTp and ORRM1 (Figure 5C). Thus, all these results suggest that RPOTp is involved in chloroplast RNA editing through its interactions with other chloroplast multiple‐site RNA editing factors.

We further compared the overlapping of RNA editing defects among *sca3‐2*, *morf2*, *morf8*, *morf9*, and *orrm1* mutants or transient‐silenced plants. The editing of *rps12*‐*i‐*58 increased in *sca3‐2* and *morf8*, while being reduced in *morf2*, *morf9*, and *orrm1*. Similarly, the decreased editing was also observed in *sca3‐2*, *morf2*, *morf8*, *morf9*, and *orrm1* for *ndhB*‐467, *ndhD‐*2, and *ndhD*‐878 sites. The editing of *ndhB*‐586, and *ndhB*‐836 was significantly reduced in *sca3‐2*, *morf2*, *morf9*, and *orrm1*. *sca3‐2*, *morf2*, and *morf9* all had significantly decreased editing at the *ndhF*‐290 and *ndhG*‐50 sites.^[^ ^13^, ^14^, ^15^ ^]^ Moreover, the editing efficiency of the *rpoC1*‐488 site significantly increased in the *sca3‐2* mutant, but it only slightly increased in *morf8/rip1* and decreased in both *morf2* and *morf9* (**Table** **1**). The overlap in the affected editing sites further supports the hypothesis that RPOTp may participate in chloroplast RNA editing through its selective interactions with chloroplast‐localized MORFs and ORRM1.

**Table 1:** Comparison of editing efficiency changes of RPOTp‐affected‐RNA editing sites between RPOTp and its interacting RNA editing factors.

Sites	∆EE				
	*sca3‐2*	*morf2/rip2* (Takenaka et al., 2012)	*morf8/rip1* (Bentolila et al., 2012)	*morf9/rip9* (Takenaka et al., 2012)	*orrm1* (Sun et al., 2015)
*rpoC1*‐488	90.62%	−17.97%^a)^	18.40%	−49.32%^a)^	0.00%
*rps12‐i*‐58	95.34%	−25.00%	162.50%	−40.00%	−99.00%
*ycf3‐*43350	307.49%	N/A	N/A	N/A	N/A
*ndhB*‐467	−31.74%	−85.00%	−75.00%	−75.00%	−95.00%
*ndhB*‐836	−40.13%	−100.00%	−3.50%	−100.00%	−95.00%
*ndhD*‐2	−78.75%	−100.00%	−55.40%	−100.00%	−57.00%
*ndhD*‐878	−22.64%	−100.00%	−70.00%	−70.00%	−97.00%
*ndhF*‐290	−23.38%	−100.00%	−3.60%	−60.00%	0.00%
*ndhG*‐50	−25.52%	−60.00%	−5.90%	−100.00%	−94.00%

∆EE = (Efficiency^Mutant^ *–* Efficiency^WT^)/Efficiency^WT^.

^a)^ Data from transient *rip2/morf2*‐silenced or *rip9/morf9*‐silenced plants (Bentolila et al., 2013).

N/A refers that the RNA editing efficiency is not reported for that site.

The PLS subfamily PPR proteins recognize distinct targets for RNA editing and bind to specific sequences near an editing site, conferring specificity to an editing complex. The site‐recognition PPR proteins for most RPOTp‐affected RNA editing sites have been identified, including OTP81 (for site *rps12*‐*i*‐58), OTP82 (for sites *ndhB*‐836 and *ndhG*‐50), OTP84 (for site *ndhF*‐290), CRR4 (for site *ndhD*‐2), CRR28 (for sites *ndhB*‐467 and *ndhD*‐878), and DOT4 (for site *rpoC1*‐488). We further assayed the interaction between RPOTp and those site‐recognition PPR proteins by LCI. However, the result indicated that RPOTp only showed interaction with OTP84 (Figure S7, Supporting Information), no interaction was observed between RPOTp and other site‐recognition PPR proteins. We speculated that RPOTp did not participate in RNA editing through site‐recognition PPR proteins.

### 2.6 RPOTp Affects the Dimerization of Chloroplast MORF Proteins

**Figure 6:**
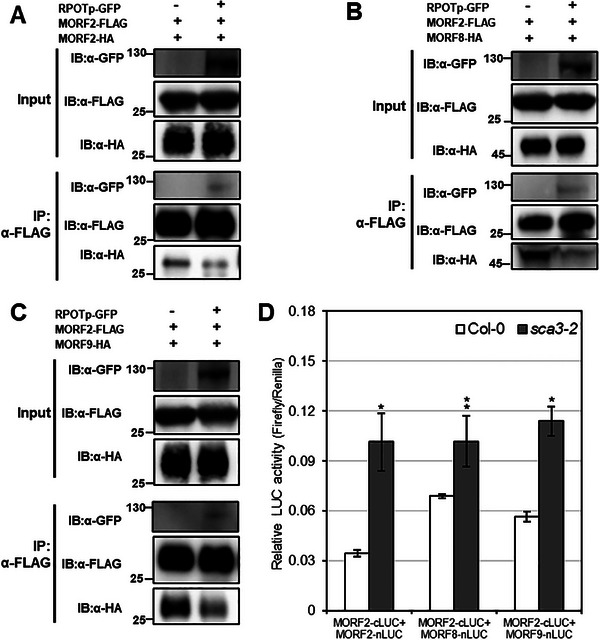
RPOTp affects the dimerization of MORF proteins. A) The Co‐IP assay shows that MORF2‐HA and RPOTp‐GFP are co‐immunoprecipitated by MORF2‐FLAG, and the presence of RPOTp reduced the immunoprecipitation of MORF2‐HA. B) The immunoprecipitation of MORF8‐HA and, C) MORF9‐HA by MORF2‐FLAG is reduced by the presence of RPOTp. RPOTp and MORF proteins are tagged with GFP, HA, or FLAG tags as indicated. Immunoprecipitation was performed with anti‐FLAG magnetic beads, and the co‐immunoprecipitated proteins were probed with the anti‐HA antibody, ‐FLAG or ‐GFP antibody. D) The firefly luciferase complementation imaging assay using Renilla luciferase as the internal control shows that the co‐expression of MORF2‐cLUC/MORF2‐nLUC, MORF2‐cLUC/MORF8‐nLUC, and MORF2‐cLUC/MORF9‐nLUC in protoplasts of the *sc3‐2* mutant has higher relative firefly luciferase (LUC) activity as compared to the corresponding co‐expression in wild‐type protoplasts. Data are the mean ± SEM from three biological replicates, and asterisks indicate statistical significance compared with the Col‐0 wild‐type protoplasts (^*^ *p* < 0.05, ^**^ *p* < 0.01) using a two‐tailed Student's *t*‐test.

To further elucidate the regulatory mechanism followed by RPOTp in affecting RNA editing efficiency in chloroplast editosomes, the dimerization of chloroplast MORF proteins was analyzed by LCI assays in the presence or absence of RPOTp. When MORF2‐nLUC was co‐expressed with MORF2‐cLUC in the presence of RPOTp, we visualized lower luminescence intensity than when MORF2‐nLUC was co‐expressed with MORF2‐cLUC in the presence of OZ1 (used as a control) in tobacco leaf epidermal cells, which indicated that the presence of RPOTp disrupted the homodimerization of MORF2 with itself (Figure S8A,B, Supporting Information). We further investigated whether RPOTp also affects the interaction of MORF2 with MORF8. MORF2‐nLUC and MORF8‐cLUC were co‐expressed in tobacco leaf epidermal cells in the presence or absence of RPOTp. Stronger luciferase activity was detected in the absence of RPOTp, while the luciferase activity was suppressed by the presence of RPOTp (Figure S8C,D, Supporting Information). Similarly, when MORF2‐nLUC was co‐expressed with MORF9‐cLUC, a relatively weaker luciferase activity compared with the control was detected in tobacco leaf epidermal cells (Figure S8E,F, Supporting Information). We further used Co‐IP assays to confirm the effect of ROPTp on dimerization of chloroplast MORF proteins, the results revealed that the precipitation of HA‐tagged MORF2 with FLAG‐tagged MORF2, which indicates the interaction of MORF2 with itself, was reduced in the presence of RPOTp (**Figure** **6**A). When HA‐tagged MORF8 was precipitated with FLAG‐tagged MORF2 in the presence of RPOTp, a weaker immunoprecipitated band of MORF8 was visualized as compared to that of in the absence of RPOTp (Figure 6B). A similar result was also observed for MORF2 and MORF9 co‐immunoprecipitation (Figure 6C). Additionally, we have also analyzed the dimerization of chloroplast MORF proteins by firefly luciferase complementation imaging assay using Renilla luciferase as the internal control in protoplasts isolated from *sca3‐2* mutant and Col‐0 wild‐type plants. When MORF2‐cLUC and MORF2‐nLUC co‐expressed, a higher relative firefly luciferase activity was observed in the *sca3‐2* protoplasts as compared to the wild‐type protoplasts (Figure 6D). Similarly, MORF2‐cLUC and MORF8‐nLUC co‐expression or MORF2‐cLUC and MORF9‐nLUC co‐expression resulted higher relative firefly luciferase activity in the *sca3‐2* mutant protoplasts as compared to the wild‐type protoplasts (Figure 6D). These results suggest that loss of RPOTp could promote or stabilize the dimerization of chloroplast MORF proteins. Taken together, these results indicate that RPOTp affects the dimerization of chloroplast MORF proteins. RPOTp, MORF2, MORF8, and MORF9 are likely to be present in a protein complex, and above‐confirmed protein interactions probably affect the stability of MORF complexes.

### 2.7 RPOTp is Required for the Expression of Chloroplast Genes

**Figure 7:**
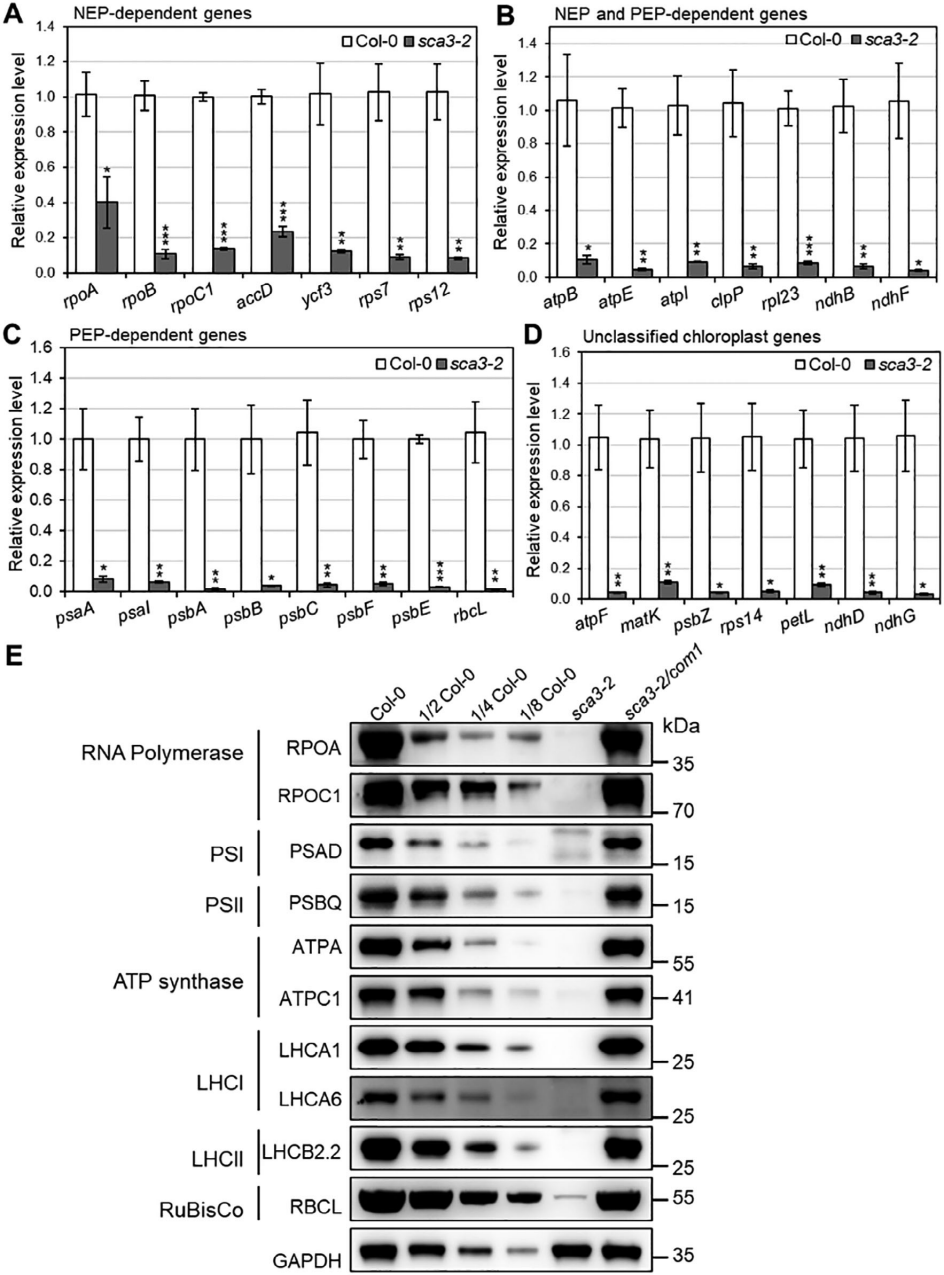
Mutant *sca3‐2* is defective in the chloroplast gene and protein expression. A–D) The *x*‐axis indicates different genes. The *y*‐axis shows the relative expression level, and expression level of each gene in Col‐0 is set to one. Data represents the mean ± SEM from three biological replicates. Asterisks represent the significance level (^*^ *p* < 0.05; ^**^ *p* < 0.01; ^***^ *p* < 0.001) compared with Col‐0 using a two‐tailed Student's *t*‐test. A) The expression levels of NEP‐dependent genes. B) The expression levels of both PEP‐ and NEP‐dependent chloroplast genes. C) The expression levels of representative PEP‐dependent genes. D) The expression level of representative unclassified chloroplast genes. E) Western blot analysis of representative chloroplast proteins in Col‐0, *sca3‐2*, and *sca3‐2/com1* plants. The lanes were loaded with a series of dilutions, as indicated. GAPDH was used as control for loading sample uniformity.

We next examined the transcript abundance of chloroplast genes in the *sca3‐2* mutant and Col‐0 wild type by qRT‐PCR. The results showed that the expression levels of NEP‐dependent genes, such as *rpoA*, *rpoB*, *rpoC1, accD*, and *ycf3*, consistently reduced in *sca3‐2* seedlings compared with Col‐0 wild type (**Figure** **7**A). Although the *ropC1* was repressed, its editing at the *rpoC1*‐488 site dramatically increased in the *sca3‐2* mutant. As for the non‐photosynthetic housekeeping class II genes transcribed by both PEP and NEP, *ndhB*, *atpB*, *atpE, atpI*, and *rpl23* also showed dramatically decreased transcript levels in the *sca3‐2* mutant (Figure 7B). The transcript levels of *atpB and rpl23* decreased by ≈80%, and the transcript levels of *atpI* and *ndhB* reduced by ≈70% in the *sca3‐2* mutant (Figure 7B). *atpE* was down‐regulated to ≈90% (Figure 7B). The expression levels of Class I PEP‐dependent genes were also drastically downregulated in the *sca3‐2* mutant. *rbcL*, which encodes the large subunit of RuBisCo, decreased to an undetectable level in the *sca3‐2* mutant (Figure 7C). The transcript levels of other PEP‐dependent genes, including *psaA*, *psaB*, and *psaI* that encode photosystem I (PSI) subunits, were also repressed by ≈85%–88% in the *sca3‐2* mutant (Figure 7C). The PEP‐transcribed‐ PSII subunit coding genes, including *psbB*, *psbC*, and *psbF*, also showed severely decreased expression levels by 90%–98% in the *sca3‐2* mutant (Figure 7C). Moreover, although the transcript levels of *rpoA*, *rpoB*, and *accD* genes significantly decreased in the *sca3‐2* mutant (Figure 7A), no significant changes in the editing efficiency of these transcripts, including *rpoA*‐200 (*rpoA*), *rpoB*‐338, *rpoB*‐551, and *rpoB*‐2432 (*rpoB*), and *accD*‐794 and *accD*‐1568 (*accD*) (Figure 2; Figure S4, Supporting Information) were observed, suggesting that there is no correlation between transcription level and RNA editing efficiency. Despite a significant decrease in the transcripts level of *rpl23*, *clpP* in the *sca3‐2* mutant (Figure 7B), the editing efficiency of *rpl23*‐89 and *clpP‐*559 was not significantly affected (Figure 2; Figure S4, Supporting Information). Furthermore, the expression levels of *psbE* and *psbF* significantly reduced in the *sca3‐2* mutant. However, the editing sites of these transcripts (*psbE‐*214 and *psbF*‐77) did not show any significant editing efficiency change in the *sca3‐2* mutant (Figure 2; Figure S4, Supporting Information). Similarly, the expression levels of *atpF*, *matK*, *psbZ*, *rps14*, and *petL* transcripts were also significantly downregulated in the *sca3‐2* mutant (Figure 7D), but no significant changes in the editing efficiency of *atpF‐*92, *matK*‐640, *psbZ*‐50, *rps14‐*80, *rps14‐*149, and *petL‐*5 sites were observed in the *sca3‐2* mutant (Figure 2; Figure S4, Supporting Information), further emphasizing that transcriptional level does not affect the RNA editing of chloroplast sites in the *sca3‐2* mutant.

We next examined protein levels of representative RNA polymerase subunits including RPOA and RPOC1, representative thylakoid complex contents including PSI (PSAD), PSII (PSBQ), light‐harvesting complex of PSI (LHCA1 and LHCA6), light‐harvesting complex of PSII (LHCB2.2), ATP synthase subunit (ATPA, ATPC1), and the large subunit of RuBisCo (RBCL) in Col‐0, *sca3‐2*, and *sca3‐2/com1* plants. RPOA and RPOC1 are the core subunits of PEP complex and are responsible for PEP activity. Western blot showed that the accumulation of RPOA and RPOC1 proteins were almost completely reduced in *sca3‐2* as compared to Col‐0 wild type. We also examined the *rpoC1* transcript and protein levels in *oz1‐1* (in which the editing of *rpoC1*‐488 is increased)^[^ ^17^ ^]^ and *dot4* (in which the editing of *rpoC1*‐488 is missing)^[^ ^36^ ^]^ mutants. The results showed that the transcription level of *rpoC1* was increased in both *oz1‐1* and *dot4* mutants as compared to the Col‐0 wild type. However, the protein level of RPOC1 in *oz1‐1* was reduced to about half of the wild type, while the protein level of RPOC1 in *dot4* was comparable to the wild type (Figure S9, Supporting Information). These results suggest that increased editing of *rpoC1*‐488 could affect the translation of RPOC1 protein, which would in turn regulate the function of PEP. The results also showed that PSAD, LHCA1, and LHCA6 of PSI, PSBQ, and LHCB2.2 of PSII were drastically reduced in *sca3‐2* compared with Col‐0 wild type and *sca3‐2/com1* (Figure 7E). The protein levels of ATP synthase subunits (ATPA and ATPC1) were also markedly reduced in the mutant plants (Figure 7E). In addition, RBCL also showed a dramatically reduced level in the *sca3‐2* mutant (Figure 7E). All these data indicate that RPOTp is indispensable for the normal accumulation of many chloroplast proteins.

## 3 Discussion

Previous studies reported that RPOTp is an important component of the NEP complex that controls chloroplast development and biogenesis by regulating the transcription of plastid genes.^[^ ^5^, ^6^ ^]^ RPOTp also functions in abiotic stress tolerance which is triggered when chloroplast functionality is perturbed by environmental stresses.^[^ ^37^ ^]^ Functional RPOTp is required for proper chloroplast biogenesis and development. However, in this study, we uncover that RPOTp is an interacting protein of MORF2 (Figure 1) and RPOTp affects the editing efficiency of nine chloroplast RNA editing sites (Figure 2). It is worth mentioning that, although the absence of different organelle RNA‐editing factors can lead to decrease in RNA editing at one or even many sites, the *sca3‐2* mutant follows a unique pattern of not only having decreased editing at six sites but also having dramatically increased editing for *rpoC1*‐488, *rps12*‐*i*‐58 and *ycf3*‐43350 (Figure 2). The role of RPOTp in RNA editing is clearly site‐specific because the editing of different RNA editing sites from the same gene are affected differently in the *sca3‐2* mutant. The *rpoC1* encodes a subunit of the PEP complex, and it is also a NEP‐dependent gene and downregulated in the *sca3‐2* mutant (Figure 7A). Thus, we speculate that increased *rpoC1* editing and decreased *rpoC1* expression both would affect the function of RPOC1, impairing the PEP activity in chloroplasts. Half of the affected sites, including *ndhB*‐467, *ndhB*‐836, *ndhD‐*2, *ndhD‐878*, *ndhF‐*290, and *ndhG*‐50, are located in genes encoding the subunits of the chloroplast NDH complex that catalyzes the cyclic electron flow^[^ ^38^ ^]^ (Figure 2). *ndhB* is a PEP‐dependent gene and encodes the B subunit of the NDH complex.^[^ ^39^ ^]^ *ndhB* gene has an important role in elevating the reactive oxygen species in chloroplasts of higher plants,^[^ ^40^, ^41^ ^]^ which may also indirectly affect the plastid genes expression. In addition, the editing at *ndhD‐*2 site is also important, in which ACG is partly edited into the translation start codon (AUG), and defects in editing at this site may lead to the abnormal conversion of the translation start codon. It is notable that RNA editing defects in *NDH* genes are also beneficial to plant immunity.^[^ ^42^ ^]^ Moreover, the editing of *rps12* and *ycf3* transcripts is also significantly affected in the *sca3‐2* mutant (Figure 2). *RPS* genes encode ribosomal proteins of the translation machinery, and proper accumulation of plastid ribosomal proteins is a prerequisite for assembling functional ribosomes and is necessary for chloroplast development. Thus, the defects in the editing of *rps* transcripts may also affect the transcription and translation of the plastid genes in *sca3‐2* by hampering the function of chloroplast ribosomes. All these results suggest that the RNA editing and ribosome biogenesis defects may also contribute to impaired plastid transcription and translation in the *sca3‐2* mutant.

It is worth noting that SIG2, SIG6, PAP2, PAP7, and RPOTp are all crucial for chloroplast gene transcription. Even though *sig2*, *sig6, pap2* and *pap7* mutants significantly changed the expression levels of most chloroplast RNA editing sites‐containing genes, our findings indicate that, except for the *rpoC1*‐488 site, the editing efficiency of most chloroplast RNA editing sites remained largely unaffected in *sig2*, *sig6*, *pap2* and *pap7* mutants (Figure 3A; Figure S6, Supporting Information), indicating that transcriptional defects do not affect the RNA editing efficiency in chloroplasts. On the contrary, the expression levels of most chloroplast RNA editing sites‐containing genes are significantly changed in the *sca3‐2* mutant (Figure 7A–D) and the editing efficiency of nine RNA editing sites are also altered in the *sca3‐2* mutant (Figure 2B,C), suggesting that the editing defects in the *sca3‐2* mutant were caused by the mutation in RPOTp itself rather than indirectly caused by the compromised chloroplast gene transcription. Furthermore, RPOTp is conserved in flowering plants (with RNA editing) and does not exist in algae (without RNA editing) (Figure S10, Supporting Information).^[^ ^3^, ^43^ ^]^ All these results indicate that RPOTp is directly involved in chloroplast RNA editing and may add a level of regulation that fine‐tunes chloroplast RNA editing specifically.

The MORF protein family constitutes an essential component of the plant organellar editosome and plays a vital role in processing almost all editing sites in chloroplasts and numerous sites in mitochondria.^[^ ^13^ ^]^ Notably, besides MORF2, RPOTp also interacts with MORF8 and MORF9 (Figure 4), as well as another chloroplast multiple‐site RNA editing factor, ORMM1 (Figure 5). The RPOTp‐affected‐RNA editing sites have selective overlapping with RPOTp‐interacting‐RNA editing factors (Table 1), indicating the selective interaction between RPOTp and chloroplast multiple‐site RNA editing factors in different editosomes in chloroplasts. MORF2, MORF8, MORF9, and ORRM1 all selectively interact with other RNA editing factors, including PPR proteins that recognize the *cis*‐elements near the target cytidine residue, and MORF proteins can form homomers or heteromers.^[^ ^20^ ^]^ Previous studies have revealed the importance of MORF dimerization in RNA editing. MORF proteins, such as MORF1 and MORF9, adopt a novel globular fold and form dimers through a hydrophobic interface. The dimerization not only stabilizes the MORF proteins but is also essential for the accurate editing process.^[^ ^44^ ^]^ Additionally, the transition from dimer to monomer may be a necessary step for MORF2 to effectively interact with PPR proteins. This transition might serve as a switch that facilitates the recruitment of MORF2 to different RNA editing complexes.^[^ ^45^ ^]^ MORF proteins also can increase the RNA binding affinity of site‐recognition PLS‐type PPR proteins for target RNAs and aid in the assembly as well as the recruitment of PPR‐E/E+ and PPR‐DYW proteins in the RNA editosomes.^[^ ^21^, ^22^, ^23^, ^24^, ^25^ ^]^ Thus, MORF proteins serve as a bridge to physically link the PPRs and other proteins to provide specificity for editing sites and cytidine deaminases. MORF2 and MORF9 also function as holdase chaperones to facilitate the folding of their client proteins and enhance their activities in chloroplasts to control various processes during chloroplast development, including RNA editing.^[^ ^46^ ^]^ However, MORF2 and MORF9 are non‐sticky proteins as previous study showed that they did not show interaction with all site‐recognition PPR proteins in RNA editosomes.^[^ ^16^ ^]^ Previously, it was reported that OsTRX z, a component of plastid transcriptionally active chromosomes (pTACs), which is essential for transcription of plastid‐encoded genes, also participates in the RNA editing of multiple chloroplast sites through its interaction with OsMORFs and regulates RNA editing by affecting the heterodimer formation of OsMORF proteins.^[^ ^47^ ^]^ The selective binding of RPOTp to chloroplast multiple‐site RNA editing factors may also affect the dimerization of MORFs and alters the stability of MORFs in chloroplast RNA editosomes (Figure 6; Figure S8, Supporting Information) to affect RNA editing efficiency at specific sites. RPOTp may also alter the chaperone activity of MORFs, or the recruitment and incorporation of other RNA editing factors into the RNA editosomes.

Our finding showed that the editing efficiency of *rpoC1*‐488, *rps12‐i*‐58, and *ycf3*‐43350 increased in the RPOTp‐defective *sca3‐2* mutant, while the other six sites had decreased RNA editing. Similar findings have also been reported in previous studies about chloroplast RNA editing factors, including MORF8/RIP1 (both *rpoC1*‐488 and *rps12‐i*‐58 had increased editing, while other chloroplast RNA editing sites showed decreased editing in the *rip1/morf8* mutant),^[^ ^13^ ^]^ OZ1 (*rpoC1*‐488 showed a significant increase of editing while other sites showed decreased editing in the *oz1* mutant),^[^ ^17^ ^]^ and PPO1 (editing of the *rpoC1*‐488 site was markedly increased while other 17 sites showed decreased editing in the *ppo1* mutant).^[^ ^16^ ^]^ It's worth noting that in wild‐type plants, the editing efficiency of *rpoC1*‐488, *rps12‐i*‐58, and *ycf3*‐43350 sites is maintained at a moderate level or nearly unedited, whereas those decreased sites are profoundly edited. We speculate that the editing activity at different sites is controlled by varied compositions of RNA editosomes. For example, different sites have different strengths or combinations of dimerization of MORF proteins. It is also possible that the moderate editing activity of *rps12‐i*‐58, *ycf3*‐43350, and *rpoC1*‐488 sites in Col‐0 wild type is maintained by a moderate dimerization strength of MORF proteins and loss of RPOTp enhances this dimerization strength, increasing the editing activity at these sites. On the contrary, for sites with decreased RNA editing efficiency, enhanced dimerization strength of MORF proteins caused by RPOTp loss may reduce the editing efficiency. Thus, it indicates that RPOTp may facilitate the correct dimerization of MORF proteins in the chloroplast editosome to affect the editing of different chloroplast sites.

**Figure 8:**
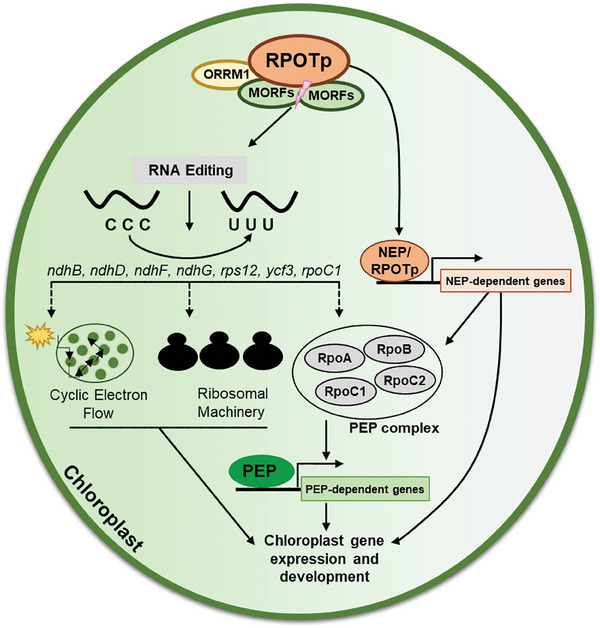
A proposed model of RPOTp in maintaining RNA editing and gene expression in chloroplasts. MORFs: multiple organelle RNA editing factor proteins; ORRM1: organelle RNA recognition motif protein 1; NEP: nucleus‐encoded RNA polymerase; PEP: plastid‐encoded RNA polymerase.

The questions of why RPOTp is recruited to affect RNA‐editing efficiency at specific sites and how this recruitment is organized deserve further study. Nevertheless, we propose a working model depicting the molecular function of RPOTp (**Figure** **8**). Nuclear‐encoded RPOTp is imported into chloroplasts, where it plays multiple roles in regulating the chloroplast gene expression. RPOTp interacts with MORFs and ORRM1 to control RNA editing of specific chloroplast genes, including NEP‐dependent *rpoC1*, which encodes an essential PEP core component. RPOTp also affects the editing of NDH complex‐coding genes such as *ndhB, ndhD*, *ndhF*, and *ndhG*, which further modulate the electron transport chain during photosynthesis. The plastid ribosomal machinery is also governed by RPOTp, as increased editing of ribosomal protein‐coding gene *rps12* is observed in the *sca3‐2* mutant. Meanwhile, the RPOTp itself functions as the nucleus‐encoded chloroplast RNA polymerase to transcribe chloroplast genes, including PEP subunit coding genes. The multiple roles of RPOTp in chloroplast gene expression are essential for chloroplast biogenesis and development.

## 4 Experimental Section

### Plant Materials and Growth Conditions

*Arabidopsis* wild‐type Col‐0 seeds were from the lab. *sca3‐2* (SALK_093884), *sig2* (SALK_045706), *sig6* (SAIL_893_C09), *oz1‐1* (SALK_032922), *dot4* (SALKseq_79621), *pap2* (SALK_128266) and *pap7* (SALK_201599) mutants were ordered from ABRC. Seeds were surface sterilized using chlorine gas for 4 h and placed on 1/2 Linsmaier and Skoog (LS) medium (Caisson, LSP03) with 0.8% micropropagation type‐1 agar (Caisson, A038). After a 4‐day stratification in the dark at 4 °C, plates were moved to long‐day conditions (16 h light/8 h dark) under 100 µmol m^−2^ s^−1^ light at 22 °C for 4 days. For collecting seeds, *Arabidopsis* plants were grown in soil under long‐day with 100 µmol m^−2^ s^−1^ light at 22 °C. *Nicotiana benthamiana* plants were grown in long‐day at 23 °C for 3–4 weeks.

### Bimolecular Fluorescence Complementation Assay (BiFC)

For BiFC assay, the coding sequence (CDS) of *SCA3* without stop codon was fused to the N‐terminus fragment of yellow fluorescent protein (nYFP) in a pCAMBIA1300‐SPYNE173 vector. Similarly, the CDS of MORF2, MORF8, MORF9, and ORRM1 without stop codon were fused to the C‐terminus fragment of YFP (cYFP) in a pCAMBIA1300‐SPYCE(M) vector. For control, the transit peptide sequence of *OTP81* was transferred to pCAMBIA‐SPYNE173 or pCAMBIA‐SPYCE(M) for N‐ or C‐terminal YFP fragment fusions targeting to chloroplasts, respectively.^[^ ^31^ ^]^ Different combinations of vectors were infiltrated into the epidermal cells of 4‐week‐old *Nicotiana benthamiana* leaves by *Agrobacterium tumefaciens* (GV3101) transformation and incubated under long‐day for 48 h at 22 °C. Interactions between target proteins were observed using a confocal microscope (Zeiss LSM 880).

### Luciferase Complementation Imaging (LCI) Assay

The LCI assay was performed in *Nicotiana benthamiana* plants as previously described.^[^ ^48^ ^]^ The CDSs of *SCA3*, *MORF2*, *MORF8*, *MORF9*, and *ORRM1* without stop codon were cloned into pCAMBIA1300‐nLUC vector for N‐terminal luciferase fragment (nLUC) fusion or modified pCAMBIA1300‐cLUC (cLUC was fused to the C‐terminal of each expressed protein) for C‐terminal luciferase fragment (cLUC) fusion.^[^ ^31^, ^49^ ^]^ Vectors were introduced into *Agrobacterium tumefaciens* and co‐infiltrated into 4‐week‐old *Nicotiana benthamiana* leaves. After 3 days of infiltration, the leaves were sprayed with luciferase substrate D‐luciferin at a final concentration of 1 mm and incubated in darkness for 10 min. The luminescence images were acquired using a CCD camera (Tanon 5200).

To construct plasmids used in the Firefly luciferase complementation imaging assay using Renilla luciferase as the internal control, the expression cassette of MORF2‐cLUC fusion fragment and the Renilla luciferase (Rluc) coding sequence were cloned into pUC19 vector to construct pUC19‐MORF2‐cLUC‐Rluc vector. Then, the fragments containing the expression cassette of MORF2‐nLUC, MORF8‐nLUC and MORF9‐nLUC were amplified and cloned into pUC19‐MORF2‐cLUC‐Rluc vector to construct the final pUC19‐MORF2‐cLUC‐MORF2‐nLUC‐Rluc, pUC19‐MORF2cLUC‐MORF8‐nLUC‐Rluc and MORF2cLUC‐MORF9‐nLUC‐Rluc vectors, respectively. In the final vectors, all target genes were driven by 35S promoter with the RBCS terminator. Transfection‐grade plasmid DNA was prepared using the QIAGEN Plasmid Maxi Kit (Qiagen, 12163). 15 µg of each plasmid was transformed into wild‐type or *sca3‐2* protoplasts isolated from Col‐0 and *sca3‐2* mutant plants following the method described previously.^[^ ^50^ ^]^ Protoplasts were then incubated under darkness at 22 °C for 16–20 h. A Dual luciferase assay was performed with the Dual‐Luciferase Reporter Gene Assay Kit (Yeasen, 11402ES60) according to the manufacturer's manual. The Renilla luciferase activity and the Firefly luciferase activity were measured using the TECAN Infinite M Plex plate reader. The data were represented as the ratio of Firefly luciferase activity to Renilla luciferase activity.

### Co‐Immunoprecipitation (Co‐IP) Assay

The CDS of *SCA3* (without stop codon) was cloned into pCAMBIA1300‐3×FLAG vector for C‐terminal 3×FLAG tag fusion, and CDSs of *MORF2*, *MORF8*, *MORF9*, *ORRM1*, and *OZ1* (without stop codon) were cloned into pCAMBIA1300‐3×HA vector for C‐terminal 3×HA tag fusion. These constructs were transformed into *Agrobacterium tumefaciens* strain GV3101. *Agrobacterium* culture containing different genes was mixed as designed and co‐infiltrated into epidermal cells of 3‐week‐old *Nicotiana benthamiana*. After 3 days, 1 g leaves were grounded and homogenized in 3 mL Plant Cell lysis buffer for Western and IP (Beyotime, P0043) with Protease and phosphatase inhibitor cocktail for plant cell and tissue extracts, 50× (Beyotime, P1055). The lysates were cleared by centrifugation at 12 000 g for 10 min at 4 °C. 240 µL of supernatant was collected as the input. The reaming supernatants were incubated with 20 µL of Anti‐FLAG® M2 magnetic beads (Millipore‐Sigma, M8823) for 2 h at 4 °C with rotation. Beads were then washed five times for 5 min with 1 mL of lysis buffer and proteins were eluted with 50 µL of 3×FLAG peptide (Beyotime, P9801) with a final concentration of 150 ng µL^−1^. The proteins were detected by immunoblotting using either an anti‐FLAG antibody (Beyotime, AF519), an anti‐HA antibody (Beyotime, AF2858), or anti‐GFP antibody (Merck, 11814460001).

### Mutant Genotyping

DNA was extracted from plants using the CTAB method. Homozygous T‐DNA mutant lines were identified using gene‐specific primers, which hybridized with the genomic sequences that flanked the insertions, in combination with the T‐DNA‐specific primer LBb1.3 (Table S2, Supporting Information). PCR was carried out using the 2 × M5 Hiper plus Taq HiFi PCR mix (with blue dye) (Mei5bio, MF002). The following thermal condition was used: 95 °C for 5 min, 36 cycles of 94 °C for 25 s, 60 °C for 30 s, and 72 °C for 1 min.

### Complementation for sca3‐2 Mutants

To obtain the complementary transgenic plants (*sca3‐2/com*) of *sca3‐2* mutants, the genomic fragment containing 1.5 kb promoter and the *SCA3* gene without stop codon was amplified by PCR using Q5® Hot Start High‐Fidelity DNA Polymerase (NEB, M0494S). The PCR fragment was inserted into the modified vector pEarleyGate101. In the final vector, *SCA3* was driven by its native promoter and fused with 3×FLAG tags at the 3'‐terminal. Then this vector was introduced into *Agrobacterium tumefaciens* strain GV3101 and transformed into *sca3‐2* mutants. Transgenic plants were selected on 1/2 LS with Basta (Sigma Aldrich) plates. T3 homozygous transgenic plants were used for further study.

### qRT‐PCR Analysis

Total RNA was isolated from whole seedlings grown under long‐day with 100 µmol m^−2^ s^−1^ light at 22 °C for 4 days using the RNAprep Pure Plant kit (Tiangen, DP432). First‐strand cDNA was synthesized using the HiScript® III 1st Strand cDNA Synthesis Kit (+gDNA wiper) with both Oligo (dT)_20_VN and random hexamer primers (Vazyme, R312‐02) added. qRT‐PCR was performed on a Bio‐Rad CFX Connect Real‐Time PCR Detection System using ChamQ Universal SYBR qPCR Master Mix (Vazyme, Q711‐02). Expression levels for all assayed genes were normalized using *PP2AA3* (*AT1G13320*)^[^ ^51^ ^]^ as the internal control. All primers used in vector construction and qRT‐PCR analysis are listed in Table S2 (Supporting Information).

### Chlorophyll Measurements

Seedlings grown under long‐day with 100 µmol m^−2^ s^−1^ light intensity at 22 °C for 4 days were harvested, measured the fresh weight, frozen in liquid nitrogen, and homogenized. Chlorophyll was extracted three times in 900 µL 80% acetone (v/v %) and cell debris was removed by centrifugation at 12 000 g for 5 min at 4 °C. Chlorophyll was measured spectrophotometrically, and levels were calculated according to the formula provided in the previous study.^[^ ^52^ ^]^

### Subcellular Localization

The CDS of *SCA3* without stop codon was amplified by PCR. The PCR fragment was subcloned into the pCAMBIA1300‐35S‐EGFP vector using the Seamless Cloning Kit (Beyotime, D7010M) to generate a fusion protein with a green fluorescent protein (GFP). The vector was transferred into *Agrobacterium tumefaciens* strain GV3101 and infiltrated into *Nicotiana benthamiana* leaves for transient expression. After 48 h of infiltration, the GFP signal was detected by a confocal microscope (Zeiss LSM880).

### Transmission Electron Microscopy (TEM)

Seedlings grown at 22 °C for 10 days under long‐day with 100 µmol m^−2^ s^−1^ light were cut into small pieces and fixed in 2.5% glutaraldehyde in phosphate buffer (pH 7.2) for 4 h at 4 °C. After fixation, the tissue was rinsed and post‐fixed overnight at 4 °C in 1% OsO_4_. After rinsing in phosphate buffer, the samples were dehydrated in an ethanol series, infiltrated with a graded series of epoxy resin in epoxy propane, and embedded in Epon 812 resin. Thin sections were stained in uranium acetate followed by lead citrate and viewed with a Hitachi H‐7650 transmission electron microscope.

### Analysis of RNA Editing

RNA extraction and cDNA synthesis were carried out as in qRT‐PCR analysis. PCR fragments containing chloroplast RNA editing sites were obtained with specific primers surrounding editing sites by RT‐PCR using OneTaq® 2× Master Mix with Standard Buffer (NEB, M0482L). PCR products were then purified and sent for Sanger sequencing. The amplifying and sequencing primers are listed in Table S3 (Supporting Information). The “C” to “T” (equal C to U in RNA) editing efficiency was measured by the relative height of the peak of the nucleotide in sequence chromatograms and calculated by the height of “T” divided by the sum of the height of “T” and “C”. Statistical significance was calculated using a two‐tailed Student's *t*‐test. For analysis of RNA editing by RNA‐sequencing, RNA samples for rRNA‐depleted RNA sequencing were prepared as same as used in RNA editing analysis. Three biological replicates were used. Ribosome RNA depletion and RNA sequencing were carried out by Novogene (Beijing, China). Fastp was used for the quality control of sequencing data.^[^ ^53^ ^]^ Raw reads were aligned to the *Arabidopsis* reference genome using Hisat2.^[^ ^54^, ^55^ ^]^ Then RNA editing efficiency was analyzed by the Chloroseq pipeline^[^ ^56^, ^57^ ^]^ and manually examined using bam files in IGV browser.^[^ ^58^ ^]^

### SDS‐PAGE and Immunoblot Analysis

Total proteins were isolated from 0.2 g of whole seedlings (grown under long‐day with 100 µmol m^−2^ s^−1^ light at 22 °C). The seedlings were homogenized in lysis buffer same as used in Co‐IP experiment and incubated at 4 °C for 30 min and centrifuged at 15 000 × g for 10 min. After the proteins were transferred onto the nitrocellulose membrane, the membrane was incubated with corresponding antibodies. Antibodies for RPOA (PHY1242), RPOC1 (PHY0381A), PSAD (PHY0056A), PSBQ (PHY2346A), LHCA1 (PHY0043A), LHCA6 (PHY0470S), and ATPA (PHY0010) were from PhytoAB, San Jose, CA, USA; ATPC1 (AS08312), and LHCB2 (AS01003) were from Agrisera, Vännäs, Sweden; GAPDH (K90002P) was from Solarbio, Beijing, China and RuBisCo (AG5359) was from Beyotime, Shanghai, China. GAPDH was used as the loading control.

### Statistical Analysis

Statistical analysis was performed using Excel. Bar graphs data were presented as fold change or percentage relative to the control, with Standard Error of the Mean (SEM) derived from three independent experiments. The Student's *t*‐test was employed for the analysis of normally distributed data. In the figures, asterisks represent levels of significance as follows: ^*^ *p* < 0.05, ^**^ *p* < 0.01, and ^***^ *p* < 0.001.

## Conflict of Interest

The authors declare no conflict of interest.

## Author Contributions

X.Z. initiated and supervised the study. X.Z., D.W., and N.A.A. designed the experiments. N.A.A. carried out most of the experiments. W.S. participated in genetic analysis. Y.Z., J.X., K.S., X.S., Y.S., and Y.J. participated in protein interaction assays and plant genotyping. N.A.A. and X.Z. analyzed the data and wrote the manuscript with input from all other authors.

## Supporting information



Supporting Information

## Data Availability

The data that support the findings of this study are available from the corresponding author upon reasonable request.
