# Supplementary material for: RNA Polymerase RPOTp is Involved in C‐to‐U RNA Editing at Multiple Sites in Arabidopsis Chloroplasts
Source: Adv Sci (Weinh). 2024 Dec 4;12(4):2405131. doi: 10.1002/advs.202405131 (PMC11789580; doi:10.1002/advs.202405131)
Supplement: Supplementary file 1 — Supporting Information [file ADVS-12-2405131-s001.docx]

**Supporting Information**

**RNA polymerase RPOTp is involved in C-to-U RNA editing at multiple sites in *Arabidopsis* chloroplasts**

Nadia Ahmed Ali, Wenjian Song, Yayi Zhang, Jiani Xing, Kexing Su, Xingxing Sun, Yujia Sun, Yizhou Jiang, Dianxing Wu, Xiaobo Zhao*


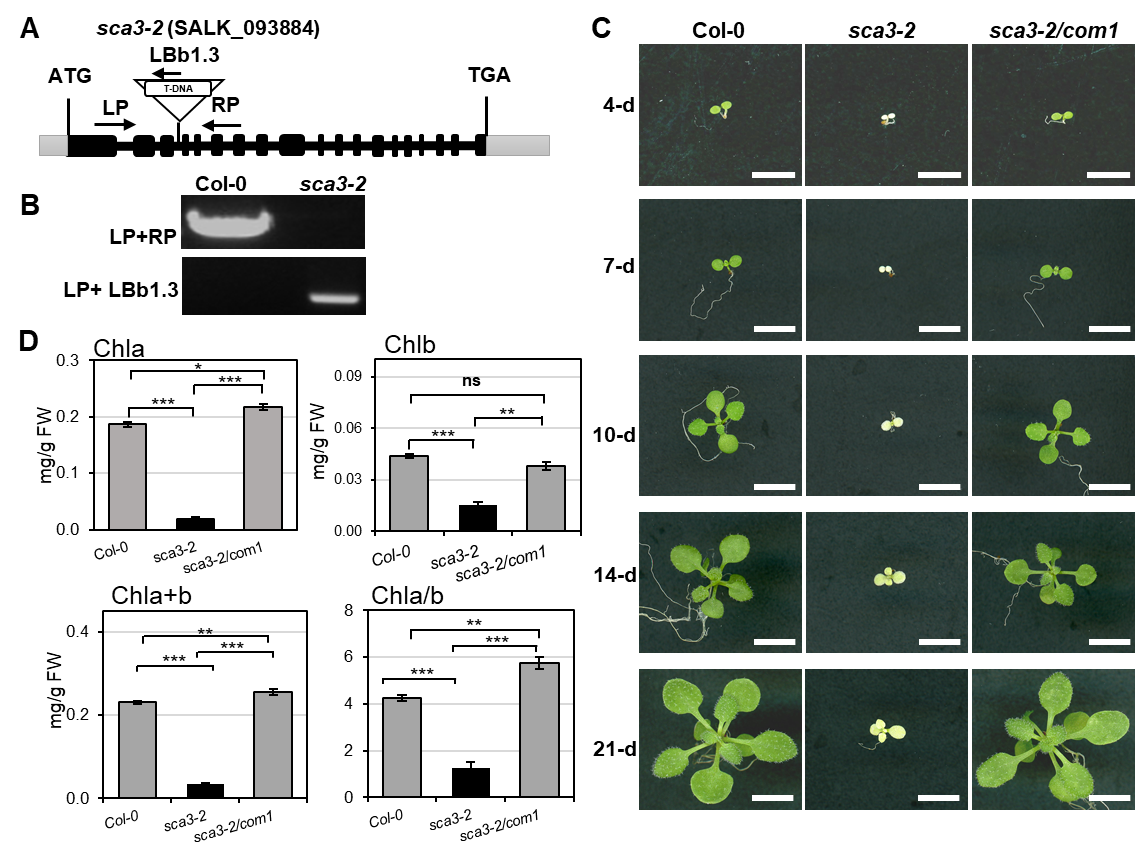


**Figure S1.** The phenotype and complementation of the *sca3-2* mutant. A) Schematic structure of the *SCA3* gene and position of T-DNA insertion in the *sca3-2* mutant. The exons are represented as black rectangles, the introns are as lines, the brown rectangles represent untranslated regions, and the T-DNA insertion is shown as a triangle in the third intron. The primers used for genotyping PCR are indicated by arrows. B) Genotyping of the T-DNA insertion in the *sca3-2* mutant. C) Phenotypic analysis of Col-0, *sca3-2* mutant, and complemented plants (*sca3-2/com1*) grown under long day conditions on 1/2LS+0.8% agar medium and pictures were taken at 4-day, 7-day, 10-day, 14-day, and 21-day. Scale bar, 0.5cm. D) Chlorophyll contents in Col-0, *sca3-2* and *sca3-2/com1* seedlings. The *y* axis is the chlorophyll levels (mg per g fresh weight). Chla: chlorophyll a; Chlb: chlorophyll b; Chla+b: total chlorophyll; Chla/b: chlorophyll a/b ratio. Data are the mean ± SEM from three biological replicates, and asterisks indicate a statistical difference compared with Col-0 wild type (**p* < 0.05, ***p* < 0.01, ****p* < 0.001, ns: not significant) using a two-tailed Student’s *t* test.


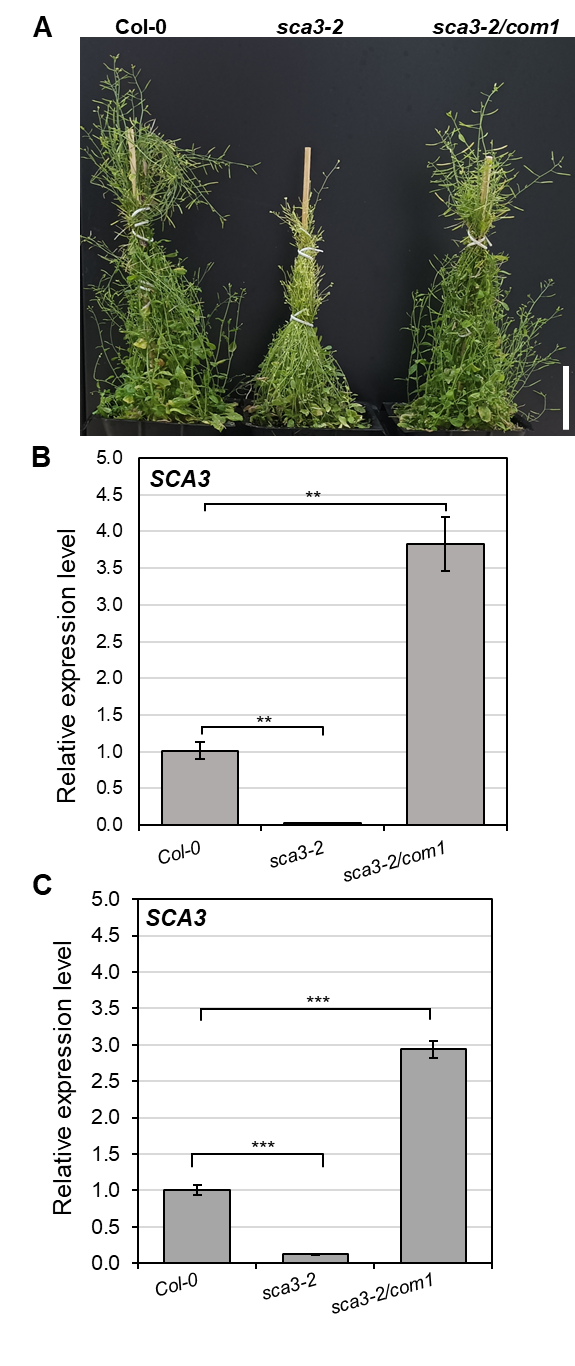


**Figure S2.** The phenotype of mature plants and expression level of *SCA3* in the *sca3-2* mutant. A) Representative images of mature (45-d-old) Col-0, *sca3-2* and *sca3-2/com1* plants. Scale bar, 6 cm. Relative expression levels of *SCA3* in *sca3-2* mutant Col-0 and *sca3-2/com1* complementary plants in B) 4-d-old plants and C) 10-d-old plants. Values are calculated from three biological replicates by normalizing against *PP2AA3*. Data are the mean ± SEM from three biological replicates, and asterisks indicate a statistical difference compared with Col-0 wild type (***p* < 0.01, ****p* < 0.001) using a two-tailed Student’s *t* test.

**
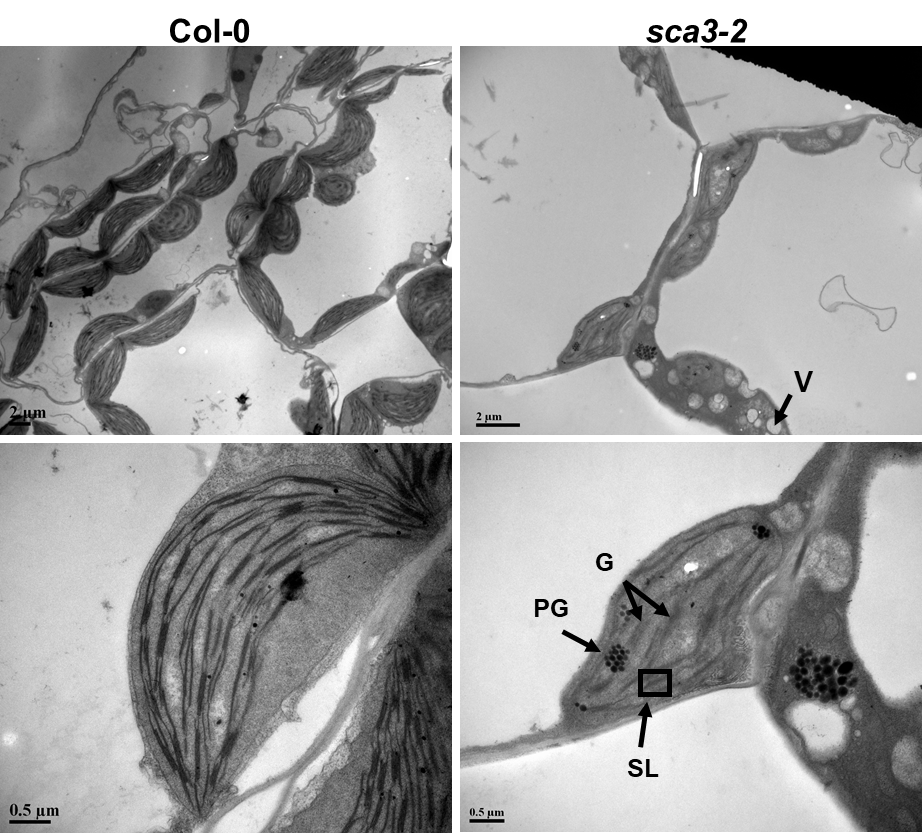
**

**Figure S3.** Transmission electron micrographs of chloroplasts in Col-0 and *sca3-2* plants. Ultrastructure of chloroplasts in Col-0 and *sca3-2* was examined under transmission electron microscopy. Plants were grown at long day conditions (16 h light/8 h dark) under 100 μmol.m^-2^.s^-1^ light intensity at 22 °C for 10 days. V: vacuole; G: grana; PG: plastoglobules; SL: stromal lamellae. Scale bars are indicated in the images.


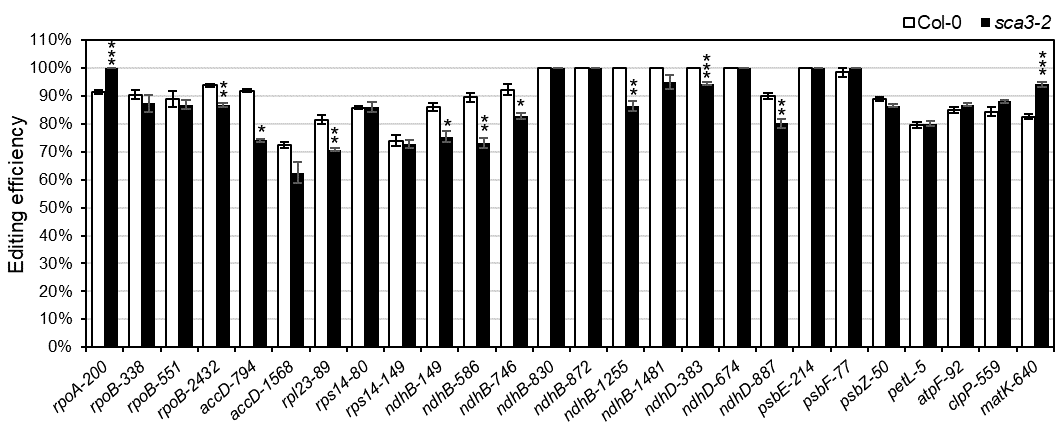


**Figure S4.** RNA editing profiles of rest chloroplast sites in 4-d-old seedlings. The sites having less than 20% decrease or increase in editing efficiency and those unaffected ones are listed. The *x* axis indicates the different RNA editing sites. The *y* axis represents the editing efficiency of each site. Data are the mean ± SEM from three biological replicates. Asterisks represent significance level **p* < 0.05, ***p* < 0.01, ****p* < 0.001 (two-tailed Student’s *t* test) compared with Col-0 wild type.

**
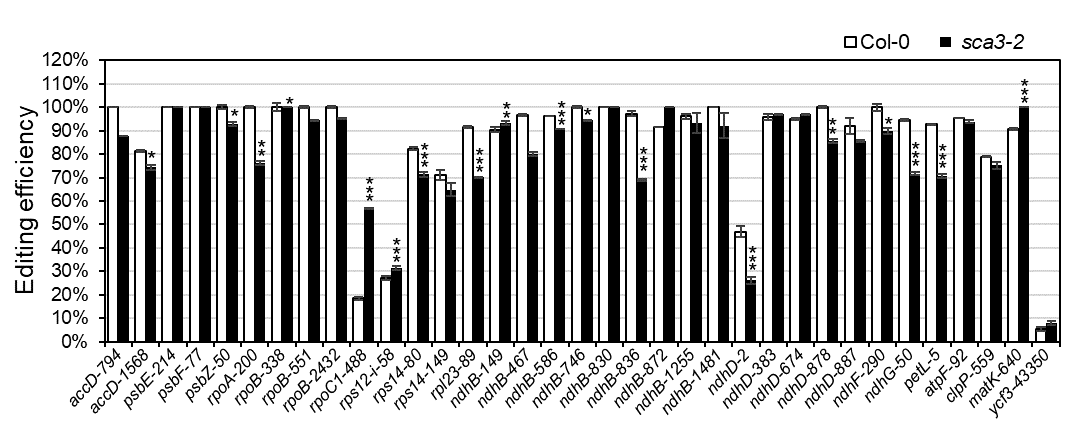
**

**Figure S5.** Chloroplast RNA editing profiles in 10-d-old plants of *sca3-2* and Col-0 wild type. The *x* axis indicates the different RNA editing sites. The *y* axis represents the editing efficiency of each site. Data are the mean ± SEM from three biological replicates. Asterisks represent significance level **p* < 0.05, ***p* < 0.01, ****p* < 0.001 (two-tailed Student’s *t* test) compared with Col-0 wild type.

**
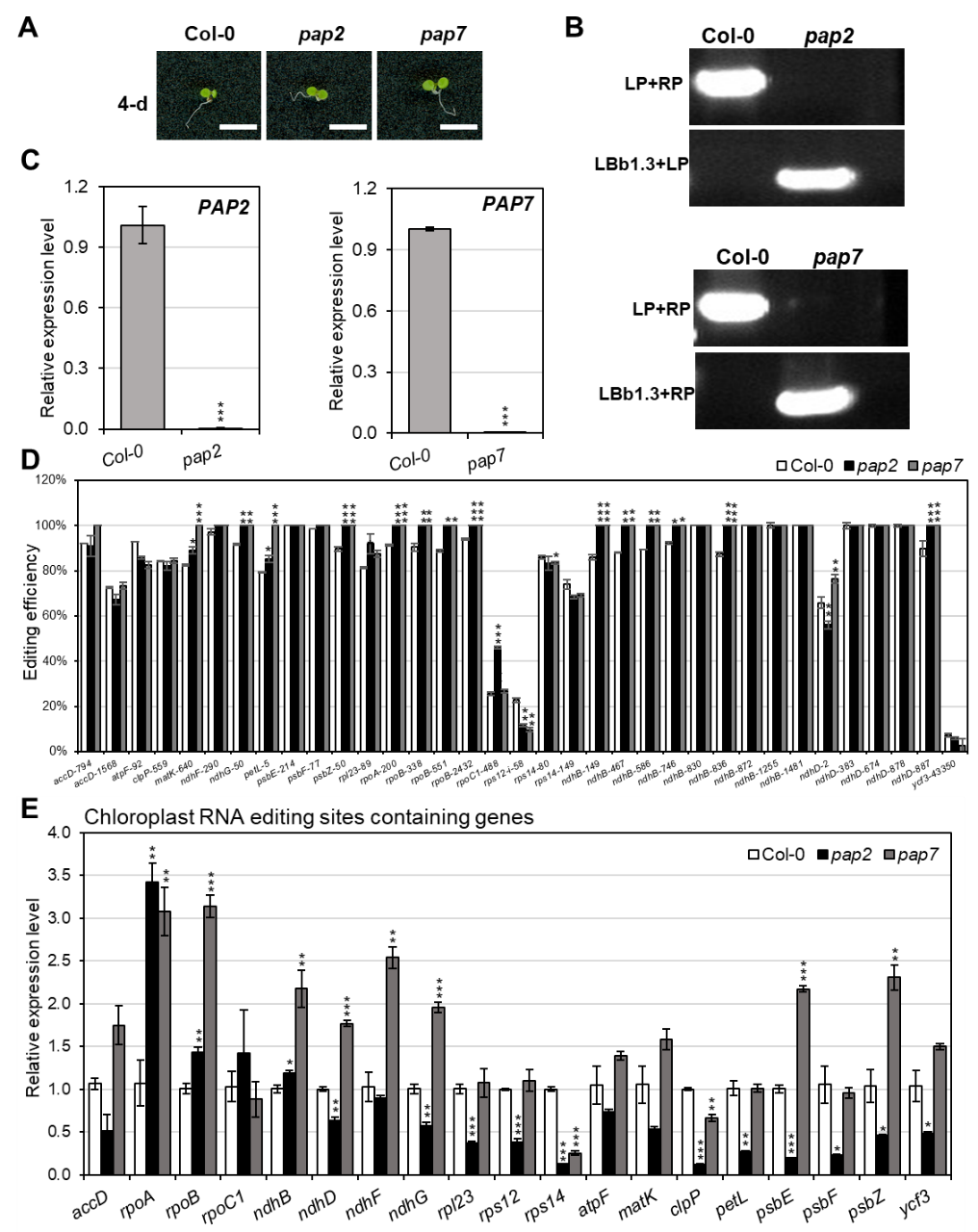
**

**Figure S6.** Chloroplast RNA editing profiles in 4-d-old seedlings of *pap2*, *pap7* and Col-0 wild type plants. A) Phenotypic analysis of *pap2* and *pap7* mutants grown under long day conditions on 1/2LS+0.8% agar medium for 4-days. Scale bar, 1cm. B) Genotyping of the T-DNA insertion in *pap2* and *pap7* mutants. C) Relative expression levels of *PAP2* and *PAP7* in 4-d-old *pap2* and *pap7* mutant plants, respectively. Values are normalized against *PP2AA3*. Data are the mean ± SEM from three biological replicates, and asterisks indicate a statistical difference compared with Col-0 wild type (****p* < 0.001) using a two-tailed Student’s *t* test. D) The *x* axis indicates the different RNA editing sites. The *y* axis represents the editing efficiency of each site. Data are the mean ± SEM from three biological replicates. Asterisks represent significance level **p* < 0.05, ***p* < 0.01, ****p* < 0.001 (two-tailed Student’s *t* test) compared with Col-0 wild type. E) Relative expression levels of chloroplast RNA editing sites-containing genes in *pap2* and *pap7* mutants as compared to Col-0 wild type plants in 4-d-old plants. Values are normalized against *PP2AA3*. Data are the mean ± SEM from three biological replicates, and asterisks indicate a statistical difference compared with Col-0 wild type (***p* < 0.01, ****p* < 0.001) using a two-tailed Student’s *t* test.

**
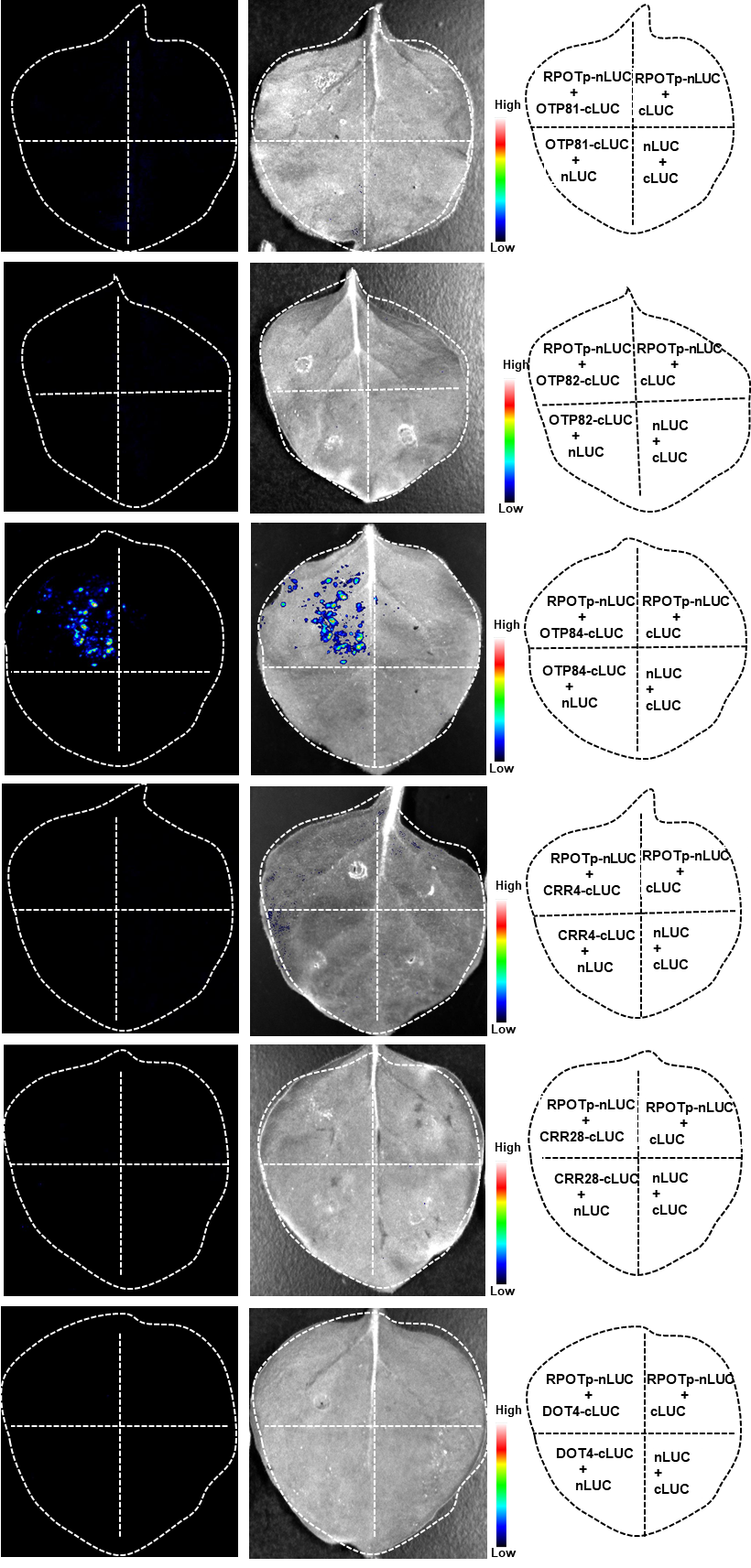
**

**Figure S7.** Interaction detection between RPOTp and site-recognition PPR proteins. LCI assays in *Nicotiana benthamiana* leaves to illustrate the interactions between RPOTp and PLS-PPRs, OTP81, OTP82, OTP84, CRR4, CRR28, and DOT4.


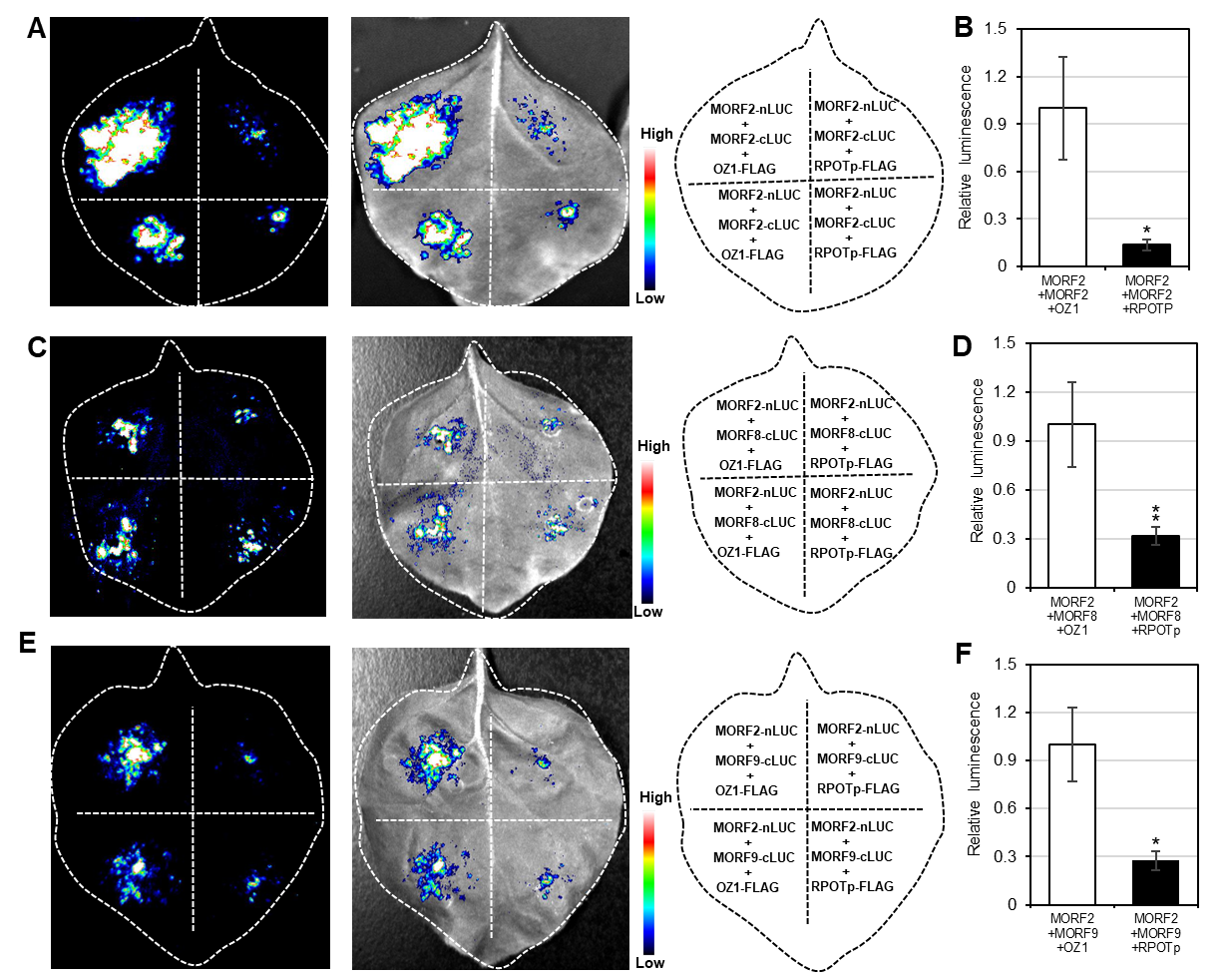


**Figure** **S8.** RPOTp affects the dimerization formation of MORF proteins. For each co-transformation combination, at least six repeats were carried out, and two representative repeats were presented. The co-transformation of MORF2-nLUC with MORF2-cLUC A), MORF2-nLUC with MORF8-cLUC C), and MORF2-nLUC with MORF9-cLUC E) in leaf epidermal cells in the presence of RPOTp-FLAG (pCAMBIA1300-RPOTp-3×FLAG used for the Co-IP assay) leads to reduced luciferase activity in the LCI assay. The co-transformations with the presence of OZ1-3×FLAG (pCAMBIA1300-OZ1-3×FLAG) were used as controls. The quantified relative luminescence strength reflecting the luciferase activity measured from six replicates of MORF2-nLUC with MORF2-cLUC B), MORF2-nLUC with MORF8-cLUC D), and MORF2-nLUC with MORF9-cLUC F). Data are the mean ± SEM from six replicates, and asterisks indicate a statistical significance compared with control (**p* < 0.05, ***p* < 0.01) using a two-tailed Student’s *t* test.


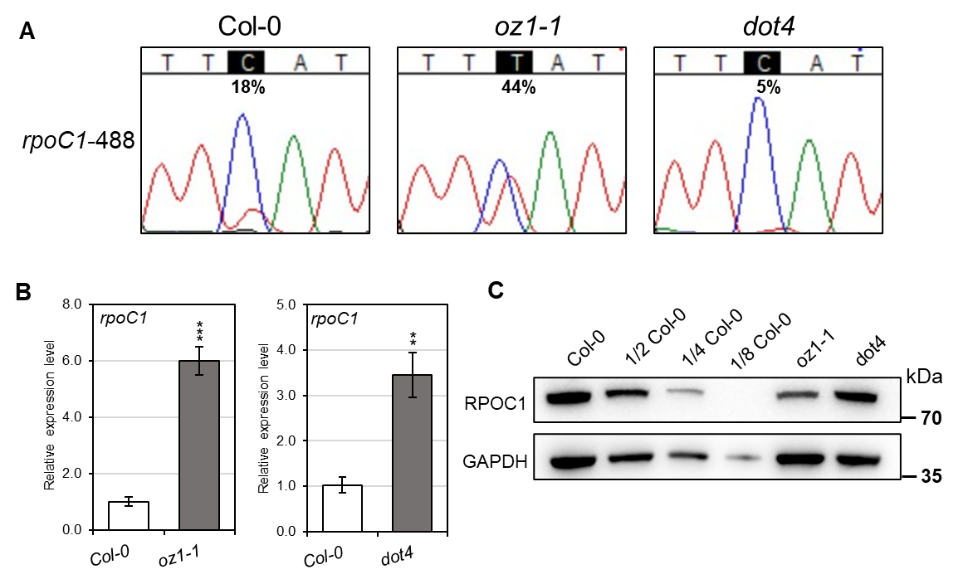


**Figure S9**. The transcript and protein level of *rpoC1* in *oz1-1* and *dot4* mutants. A) Sequencing chromatogram show the RNA editing efficiency (percentage) of *rpoC1-*488 in *oz1-1* and *dot4* mutants compared with the Col-0 wild type. The edited sites are highlighted by dark blocks, and the calculated C to T (equal C to U in RNA) editing efficiencies are labeled. B) The relative transcript level of *rpoC1* in 10-d-old *oz1-1* and *dot4* mutants as compared to the Col-0 wild type. Values are calculated from three biological replicates by normalizing against *PP2AA3*. Data are the mean ± SEM from three biological replicates, and asterisks indicate a statistical difference compared with Col-0 wild type (***p* < 0.01, ****p* < 0.001) using a two-tailed Student’s *t* test. C) Western blot analysis of RPOC1 protein in Col-0, *oz1-1*, and *dot4* plants. The lanes were loaded with a series of dilutions, as indicated. GAPDH was used as control for loading sample uniformity.

**
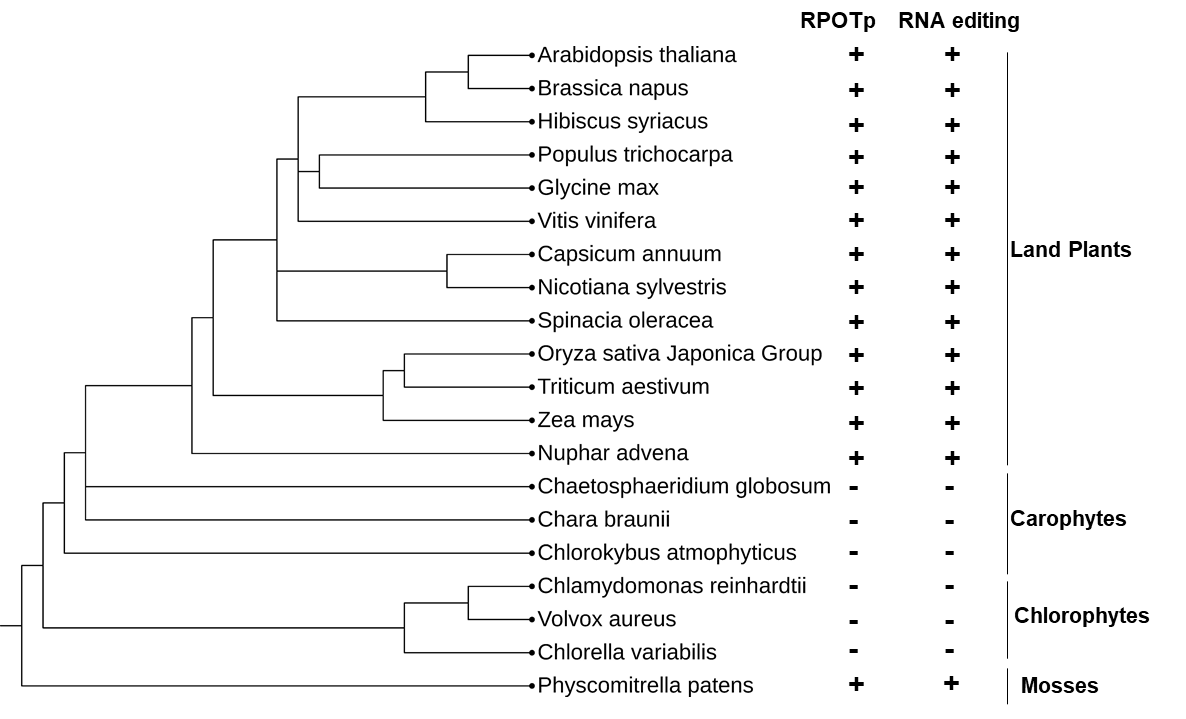
**

**Figure S10**. Distribution of RPOTp protein across different plant lineages. The schematic tree on the left shows the evolutionary relationships. “+” means exist while “-” indicates not exist. In algae, no RNA editing has been reported to date and no RPOTp exists as well.

**Table S1. Chloroplast RNA editing profiles in the *sca3-2* mutant and Col-0 wild type revealed by Chloroseq analysis.**

| Editing site | **Editing efficiency (%)** | | | | | **Editing efficiency (%)** | | | | | *p* value |
| --- | --- | --- | --- | --- | --- | --- | --- | --- | --- | --- | --- |
|  | Col-0*-*R1 | Col-0*-*R2 | Col-0*-*R3 | Col-0-Mean | Col-0-SEM | *sca3-2-*R1 | *sca3-2-*R2 | *sca3-2-*R3 | *sca3-2*-Mean | *sca3-2*-SEM |  |
| matK-640 | 83.76 | 82.80 | 82.94 | 83.17 | 0.2981 | 93.01 | 93.41 | 94.95 | 93.79 | 0.5922 | 8.88108E-05 |
| atpF-92 | 88.19 | 88.00 | 87.23 | 87.81 | 0.2943 | 93.46 | 92.44 | 93.95 | 93.29 | 0.4467 | 0.000512109 |
| atpH-3’UTR-13210 | 2.31 | 1.30 | 1.64 | 1.75 | 0.2982 | 2.41 | 2.97 | 3.55 | 2.97 | 0.3287 | 0.050726125 |
| rpoC1-488 | 45.49 | 46.17 | 48.53 | 46.73 | 0.0092 | 70.24 | 76.87 | 77.33 | 76.87 | 4.0356 | 0.000341543 |
| rpoB-2432 | 89.10 | 82.85 | 86.41 | 86.12 | 1.8104 | 82.60 | 90.84 | 91.87 | 88.44 | 2.9322 | 0.538271702 |
| rpoB-551 | 88.43 | 86.09 | 90.70 | 88.41 | 1.3315 | 78.72 | 82.24 | 88.09 | 83.02 | 2.7329 | 0.151002874 |
| rpoB-338 | 86.56 | 86.56 | 90.15 | 87.75 | 1.1955 | 91.72 | 88.94 | 95.13 | 91.93 | 1.7893 | 0.124190342 |
| psbZ-50 | 91.11 | 91.09 | 90.68 | 90.96 | 0.1417 | 94.18 | 95.34 | 95.42 | 94.98 | 0.4005 | 0.000696738 |
| rps14-149 | 88.79 | 88.98 | 88.27 | 88.68 | 0.2131 | 97.19 | 96.73 | 96.89 | 96.94 | 0.1362 | 5.24673E-06 |
| rps14-80 | 94.35 | 94.40 | 94.60 | 94.45 | 0.0742 | 96.18 | 96.08 | 96.72 | 96.33 | 0.1979 | 0.00089479 |
| ycf3-43350 | 5.59 | 4.37 | 4.46 | 4.81 | 0.3936 | 36.31 | 33.10 | 36.35 | 35.25 | 1.0761 | 1.19233E-05 |
| N_rps4_UTR | 1.62 | 1.54 | 1.57 | 1.58 | 0.0217 | 4.63 | 4.48 | 4.25 | 4.45 | 0.1078 | 1.27313E-05 |
| N_ndhK_ndhJ | 2.08 | 1.89 | 1.86 | 1.94 | 0.0690 | 0.51 | 0.35 | 0.73 | 0.53 | 0.1102 | 0.000404752 |
| accD-794 | 94.52 | 94.83 | 94.74 | 94.69 | 0.0896 | 95.29 | 95.29 | 96.70 | 95.76 | 0.4676 | 0.088652643 |
| accD-1568 | 79.82 | 79.10 | 80.73 | 79.88 | 0.4707 | 91.79 | 90.95 | 87.86 | 90.20 | 1.1973 | 0.00131165 |
| psbF-77 | 92.88 | 92.74 | 93.28 | 92.97 | 0.1621 | 98.41 | 98.18 | 98.57 | 98.39 | 0.1146 | 1.06932E-05 |
| psbE-214 | 99.60 | 99.53 | 99.68 | 99.60 | 0.0427 | 99.57 | 99.40 | 99.68 | 99.55 | 0.0801 | 0.585819439 |
| petL-5 | 79.05 | 82.06 | 76.43 | 79.18 | 1.6240 | 85.02 | 87.03 | 86.12 | 86.06 | 0.5814 | 0.016308681 |
| N_rps18_UTR | 0.00 | 3.47 | 0.00 | 1.15 | 1.1594 | 0.92 | 0.00 | 1.56 | 0.82 | 0.4536 | 0.804094023 |
| rps12-i-58 | 22.33 | 17.70 | 23.64 | 21.22 | 1.8028 | 55.23 | 53.60 | 54.24 | 54.36 | 0.4744 | 5.88616E-05 |
| clpP-559 | 84.27 | 86.53 | 85.74 | 85.51 | 0.6612 | 95.95 | 96.85 | 97.27 | 96.69 | 0.3882 | 0.000128678 |
| rpoA-200 | 86.02 | 85.44 | 86.99 | 86.15 | 0.4517 | 78.36 | 87.80 | 85.94 | 84.03 | 2.8894 | 0.509470255 |
| rpl23-89 | 81.60 | 80.35 | 81.92 | 81.29 | 0.4795 | 78.82 | 79.11 | 77.67 | 78.53 | 0.4389 | 0.013208825 |
| ycf2-as-91535 | 3.80 | 2.43 | 2.48 | 2.91 | 0.4490 | 9.63 | 4.25 | 4.28 | 6.05 | 1.7894 | 0.163147273 |
| N_ndhB_UTR | 0.00 | 0.00 | 0.00 | 0.00 | 0.0000 | 0.00 | 0.00 | 0.00 | 0.00 | 0.0000 | #DIV/0! |
| ndhB-1481 | 92.20 | 90.12 | 90.08 | 90.80 | 0.7004 | 86.63 | 89.59 | 88.08 | 88.10 | 0.8543 | 0.07102147 |
| ndhB-1255 | 96.81 | 97.05 | 96.77 | 96.88 | 0.0877 | 90.37 | 92.35 | 92.44 | 91.72 | 0.6739 | 0.001616023 |
| ndhB-872 | 84.69 | 81.73 | 83.28 | 83.23 | 0.8544 | 77.53 | 75.95 | 78.40 | 77.29 | 0.7162 | 0.005968762 |
| ndhB-836 | 78.03 | 77.41 | 75.02 | 76.82 | 0.0092 | 41.38 | 41.81 | 43.43 | 42.21 | 0.0063 | 0.000269959 |
| ndhB-830 | 75.81 | 74.87 | 75.43 | 75.37 | 0.2736 | 60.65 | 66.25 | 71.16 | 66.02 | 3.0361 | 0.037409036 |
| ndhB-746 | 91.55 | 90.45 | 90.24 | 90.75 | 0.0041 | 85.33 | 85.55 | 85.80 | 85.56 | 0.0014 | 0.002889912 |
| N_ndhB_2 | 0.67 | 0.65 | 0.64 | 0.65 | 0.0104 | 1.82 | 1.00 | 0.75 | 1.19 | 0.3212 | 0.170756448 |
| N_ndhB_1 | 2.03 | 2.27 | 2.01 | 2.10 | 0.0835 | 2.33 | 0.35 | 2.05 | 1.58 | 0.6168 | 0.443606489 |
| ndhB-586 | 91.57 | 90.27 | 89.01 | 90.28 | 0.7410 | 83.49 | 81.17 | 84.60 | 83.08 | 1.0113 | 0.004555985 |
| ndhB-467 | 90.32 | 88.40 | 88.66 | 89.13 | 0.0060 | 75.08 | 70.02 | 72.64 | 72.58 | 0.0146 | 0.000529002 |
| ndhB-149 | 92.82 | 92.78 | 93.72 | 93.11 | 0.3081 | 90.91 | 91.97 | 91.14 | 91.34 | 0.3197 | 0.016398404 |
| ndhF-290 | 80.29 | 78.65 | 80.23 | 79.73 | 0.0054 | 46.71 | 47.50 | 49.93 | 48.05 | 0.0097 | 8.87145E-06 |
| ndhD-887 | 95.41 | 95.11 | 94.70 | 95.07 | 0.2065 | 94.02 | 94.95 | 93.73 | 94.23 | 0.3675 | 0.117268865 |
| ndhD-878 | 89.21 | 88.93 | 88.12 | 88.75 | 0.0033 | 64.29 | 61.86 | 66.32 | 77.55 | 0.1480 | 1.27223E-05 |
| ndhD-674 | 97.01 | 97.23 | 97.11 | 97.12 | 0.0622 | 97.52 | 97.37 | 97.15 | 64.16 | 0.0129 | 0.136127873 |
| ndhD-383 | 95.88 | 95.94 | 95.53 | 95.78 | 0.1287 | 93.54 | 94.67 | 92.83 | 93.68 | 0.5337 | 0.018619212 |
| ndhD-2 | 61.64 | 61.54 | 63.67 | 62.28 | 0.6958 | 20.94 | 21.03 | 21.24 | 21.07 | 0.0884 | 5.02255E-07 |
| ndhG-50 | 85.83 | 85.25 | 83.72 | 84.93 | 0.6282 | 50.00 | 50.86 | 53.34 | 51.40 | 1.0026 | 9.22229E-06 |

**Table S2. List of all the primers used in vector construction and qRT-PCR analysis.**

| **Primer Name** | **Vectors** | **Oligonucleotides Sequence** | **Assay** |
| --- | --- | --- | --- |
| RPOTp-F |  | ATCAGCCCAGAGCCTTTCTAC | Genotyping of T-DNA insertion |
| RPOTp-R |  | GTTGAGTCGTGGTTTTGGTTG | Genotyping of T-DNA insertion |
| PAP2-F |  | TCACACAAAGCACTAGCATCG | Genotyping of T-DNA insertion |
| PAP2-R |  | AGAATCTCCTATCGCGAAAGC | Genotyping of T-DNA insertion |
| PAP7-F |  | AGTTTTTCTGGTCTGCTGCAG | Genotyping of T-DNA insertion |
| PAP7-R |  | TTGTTTGGTTTGGACCGTAAC | Genotyping of T-DNA insertion |
| LBb1.3 |  | ATTTTGCCGATTTCGGAAC | Genotyping of T-DNA insertion |
| RPOTp-3×FLAG-gF | pEarleyGate 101 | GAGGCAAGAGCAGCAGCTGACGCGTTTAGCCAACTCTTCTCTC | Complementation of RPOTp |
| RPOTp-3×FLAG-gR | pEarleyGate 101 | CATCCTTGTAATCGAGCTCCCTAGGGTTGAAGAAGTACTGTGATTTG | Complementation of RPOTp |
| RPOTp-GFP-F | pCAMBIA1300 | CAGCTATGACCATGATTACGAATTCAGCTTGCATGCCTGCAGG | Subcellular Localization |
| RPOTp-GFP-R | pCAMBIA1300 | TAAAACGACGGCCAGTGCCAAGCTTCCGATCTAGTAACATAGATGACACCG | Subcellular Localization |
| RPOTp-3×FLAG-F | pCAMBIA1300 | CAGGCCTGGCGCGCCACTAGTATGGCTTCCGCTGCGGCG | Co-IP |
| RPOTp-3×FLAG-R | pCAMBIA1300 | CTTGTAATCCCTAGGGGTACCGTTGAAGAAGTACTGTGATTTGAGAACTT | Co-IP |
| MORF2-3×HA-F | pCAMBIA1300 | CGGACTAGTATGGCTTTGCCTTTGTCTGGC | Co-IP |
| MORF2-3×HA-R | pCAMBIA1300 | ATCGTATGGGTAAGCGTAATCTGGAACATCGTATGGGTAGGTACCCCTA  GGCTCGAGACGCGTTCTTGTGTTTTCTCTGCGGCG | Co-IP |
| MORF8-3×HA-F | pCAMBIA1300 | GGCTCAGGCCTGGCGCGCCACTAGTGAGCTCGGTACCATGGCG | Co-IP |
| MORF8-3×HA-R | pCAMBIA1300 | AACATCGTATGGGTAGGTACCGTCGACACCCTGGTAGGG | Co-IP |
| MORF9-3×HA-F | pCAMBIA1300 | GGCTCAGGCCTGGCGCGCCACTAGTGAGCTCGGTACCATGGCTTC | Co-IP |
| MORF9-3×HA-R | pCAMBIA1300 | AACATCGTATGGGTAGGTACCGTCGACAGAGGAATCAGAGG | Co-IP |
| ORRM1-3×HA-F | pCAMBIA1300 | GGCTCAGGCCTGGCGCGCCACTAGTATGGAAGCTCTTATTGCTTC | Co-IP |
| ORRM1-3×HA-R | pCAMBIA1300 | AACATCGTATGGGTAGGTACCGAGCCCGAAACTTGGTTG | Co-IP |
| OZ1-3×HA-F | pCAMBIA1300 | GGCTCAGGCCTGGCGCGCCACTAGTATGAACAACTCCACCAGAC | Co-IP |
| OZ1-3×HA-R | pCAMBIA1300 | AACATCGTATGGGTAGGTACCTTTATCTCCTTTACCAGTGG | Co-IP |
| RPOTp-nLUC-F | pCAMBIA1300 | GAGAACACGGGGGACGAGCTCATGGCTTCCGCTGCGGCG | LCI |
| RPOTp-nLUC-R | pCAMBIA1300 | CGCGTACGAGATCTGGTCGACGTTGAAGAAGTACTGTGATTTGAGAACTT | LCI |
| MORF2-cLUC-F | pCAMBIA1300 | CGGAGCTCATGGCTTTGCCTTTGTCTGG | LCI |
| MORF2-cLUC-R | pCAMBIA1300 | ACGCGTCGACTCTTGTGTTTTCTCTGCGGC | LCI |
| MORF8-cLUC-F | pCAMBIA1300 | GAGAACACGGGGGACGAGCTCATGGCGACGCATACCATTTC | LCI |
| MORF8-cLUC-R | pCAMBIA1300 | GGGACGCGTACGAGATCTGGTCGACACCCTGGTAGGGGTTGCC | LCI |
| MORF9-cLUC-F | pCAMBIA1300 | GAGAACACGGGGGACGAGCTCATGGCTTCCTTCACAACAAC | LCI |
| MORF9-cLUC-R | pCAMBIA1300 | GGGACGCGTACGAGATCTGGTCGACAGAGGAATCAGAGGCTGC | LCI |
| ORRM1-cLUC-F | pCAMBIA1300 | GAGAACACGGGGGACGAGCTCATGGAAGCTCTTATTGCTTC | LCI |
| ORRM1-cLUC-R | pCAMBIA1300 | GGGACGCGTACGAGATCTGGTCGACGAGCCCGAAACTTGGTTG | LCI |
| RPOTp-NE-F | pCAMBIA1300 | CAGCTATGACCATGATTACGAATTCAGCTTGCATGCCTGCAGG | BiFC |
| RPOTp-NE-R | pCAMBIA1300 | TAAAACGACGGCCAGTGCCAAGCTTCCGATCTAGTAACATAGATGACACCG | BiFC |
| MORF2-CE-F | pCAMBIA1300 | GGCTCAGGCCTGGCGCGCCACTAGTATGGCTTTGCCTTTGTCTG | BiFC |
| MORF2-CE-R | pCAMBIA1300 | GTACATCCCGGGAGCGGTACCTGTGTTTTCTCTGCGGCG | BiFC |
| MORF8-CE-F | pCAMBIA1300 | AGCTATGACCATGATTACGGAATTCAGCTTGCATGCCTGCAGG | BiFC |
| MORF8-CE-R | pCAMBIA1300 | CGACGGCCAGTGCCAAGCTAAGCTTCCGATCTAGTAACATAGATGACACCG | BiFC |
| MORF9-CE-F | pCAMBIA1300 | AGCTATGACCATGATTACGGAATTCAGCTTGCATGCCTGCAGG | BiFC |
| MORF9-CE-R | pCAMBIA1300 | CGACGGCCAGTGCCAAGCTAAGCTTCCGATCTAGTAACATAGATGACACCG | BiFC |
| ORRM1-CE-F | pCAMBIA1300 | GGCTCAGGCCTGGCGCGCCACTAGTATGGAAGCTCTTATTGCTTC | BiFC |
| ORRM1-CE-R | pCAMBIA1300 | GTACATCCCGGGAGCGGTACCGAGCCCGAAACTTGGTTG | BiFC |
| MORF2-cLUC-MORF2-nLUC-F | pUC19 | TTGTAAAACGACGGCCAGTGAATTCGGTCCCCAGATTAGCCTTTTC | LCI |
| MORF2-cLUC-MORF2-nLUC-R | pUC19 | AAAGGCTAATCTGGGGACCGTTGATGCATGTTGTCAATCAATTG | LCI |
| MORF2-cLUC-MORF9-nLUC-F | pUC19 | TTGTAAAACGACGGCCAGTGAATTCGGTCCCCAGATTAGCCTTTTC | LCI |
| MORF2-cLUC-MORF9-nLUC-R | pUC19 | AAAGGCTAATCTGGGGACCGTTGATGCATGTTGTCAATCAATTG | LCI |
| MORF2-cLUC-MORF8-nLUC-F | pUC19 | TTGTAAAACGACGGCCAGTGAATTCGGTCCCCAGATTAGCCTTTTC | LCI |
| MORF2-cLUC-MORF8-nLUC-R | pUC19 | AAAGGCTAATCTGGGGACCGTTGATGCATGTTGTCAATCAATTG | LCI |
| MORF2-cLUC-MORF2-nLUC-RLUC-F | pUC19 | ATTGTACTGAGAGTGCACCATATGGGTCCCCAGATTAGCCTTTTC | LCI |
| MORF2-cLUC-MORF2-nLUC-RLUC-R | pUC19 | GTGCGGTATTTCACACCGCATTGATGCATGTTGTCAATCAATTG | LCI |
| MORF2-cLUC-MORF9-nLUC-RLUC-F | pUC19 | ATTGTACTGAGAGTGCACCATATGGGTCCCCAGATTAGCCTTTTC | LCI |
| MORF2-cLUC-MORF9-nLUC-RLUC-R | pUC19 | GTGCGGTATTTCACACCGCATTGATGCATGTTGTCAATCAATTG | LCI |
| MORF2-cLUC-MORF8-nLUC-RLUC-F | pUC19 | TTGTAAAACGACGGCCAGTGAATTCGGTCCCCAGATTAGCCTTTTC | LCI |
| MORF2-cLUC-MORF8-nLUC-RLUC-R | pUC19 | AAAGGCTAATCTGGGGACCGTTGATGCATGTTGTCAATCAATTG | LCI |
| RPOTp-qPCR-F |  | GTCTGCTAGAACTATTGA | qRT-PCR |
| RPOTp-qPCR-R |  | CACTAACATCGGAACATAAG | qRT-PCR |
| PAP2-qPCR-F |  | TTCAAGCAGTAGAGTCAA | qRT-PCR |
| PAP2-qPCR-R |  | GGTCTAACAATCCATCAA | qRT-PCR |
| PAP7-qPCR-F |  | TTCATACTTGGTGCTCACT | qRT-PCR |
| PAP7-qPCR-R |  | GCGTTTGTTGGCTAATCA | qRT-PCR |
| RPOA-qPCR-F |  | AACATAGCAGGTATTCAA | qRT-PCR |
| RPOA-qPCR-R |  | TAGGATAACTTCTGTCTTC | qRT-PCR |
| RPOB-qPCR-F |  | GCAGGTTAGAATTAGAGATTGATA | qRT-PCR |
| RPOB-qPCR-R |  | GGGTAGCAAACATTCTCTAGAAT | qRT-PCR |
| RPOC1-qPCR-F |  | CCTAGTTATATTGCGAATCTT | qRT-PCR |
| RPOC1-qPCR-R |  | AAAGTGGGATGCTGTATT | qRT-PCR |
| ACCD-qPCR-F |  | CAATTCTTACATCTCCTACTA | qRT-PCR |
| ACCD-qPCR-R |  | GGTTCGGCAATAATGATA | qRT-PCR |
| YCF3-qPCR-F |  | GCCTTATCCATACAAGTAATG | qRT-PCR |
| YCF3-qPCR-R |  | GTTCTCCACGGTAATGAC | qRT-PCR |
| PSAA-qPCR-F |  | TTATGGTAAGAGACTATGA | qRT-PCR |
| PSAA-qPCR-R |  | GACTGGTTGTAATTGTAT | qRT-PCR |
| PSBA-qPCR-F |  | CTAATGAAGGTTACAGATT | qRT-PCR |
| PSBA-qPCR-R |  | TAGCACGGTTAATAATATC | qRT-PCR |
| PSAI-qPCR-F |  | CTTTCAATAACTTACCCTCTA | qRT-PCR |
| PSAI-qPCR-R |  | GAAGAAATAAAGAAGCCATT | qRT-PCR |
| PSBB-qPCR-F |  | CTATTCCATCTTAGTGTTC | qRT-PCR |
| PSBB-qPCR-R |  | TTCTTGTTGAAAGTATCC | qRT-PCR |
| PSBC-qPCR-F |  | GAGAAGTTATAGACACCTT | qRT-PCR |
| PSBC-qPCR-R |  | GAGCCTTGAATACTAGAA | qRT-PCR |
| PSBF-qPCR-F |  | TGGTTGGCTGTTCATGGACTA | qRT-PCR |
| PSBF-qPCR-R |  | TTGGATGAACTGCATTGCTGAT | qRT-PCR |
| ATPB-qPCR-R |  | CAACATCTCCTATCCATA | qRT-PCR |
| ATPB-qPCR-R |  | TTCATTCATCTGACCATA | qRT-PCR |
| ATPE-qPCR-F |  | AATTGTTTGGGATTCAGA | qRT-PCR |
| ATPE-qPCR-R |  | AATGTCACTATTCTTCTCT | qRT-PCR |
| ATPI-qPCR-F |  | CAGCACCAACGAATGATA | qRT-PCR |
| ATPI-qPCR-R |  | ACAAGAACAACAACGACTA | qRT-PCR |
| NDHB.2-qPCR-F |  | TATGCTCCTACCTATTATC | qRT-PCR |
| NDHB.2-qPCR-R |  | CCTACAGTGATGAATATAAG | qRT-PCR |
| RPS12B-qPCR-F |  | GCCATAATTTACAAGAACATTC | qRT-PCR |
| RPS12B-qPCR-R |  | TTACTCCGACAGCATCTA | qRT-PCR |
| RPS7-qPCR-F |  | AAAGACAGAAACAAATCC | qRT-PCR |
| RPS7-qPCR-R |  | CCTAATAACCAACGAATG | qRT-PCR |
| RPL23-qPCR-F |  | TTGGTGTCAAGGTAATAG | qRT-PCR |
| RPL23-qPCR-R |  | AGAGGTGGAATAGAATAAC | qRT-PCR |
| RBLC-qPCR-F |  | AAGATGATGAGAATGTGAA | qRT-PCR |
| RBLC-qPCR-R |  | GTGGATGTGAAGAAGTAG | qRT-PCR |
| ATPF-qPCR-F |  | AAGAGAGGAATGGACGTGG | qRT-PCR |
| ATPF-qPCR-R |  | GCGGATTTATGGATAGGAGA | qRT-PCR |
| CLPP-qPCR-F |  | TGACATATACAACCGACTT | qRT-PCR |
| CLPP-qPCR-R |  | TAGCCATTCCAGATATTACC | qRT-PCR |
| PETL-qPCR-F |  | TTTCGGTTTTCTACTAGCAGCTTT | qRT-PCR |
| PETL qPCR-R |  | TGCTTAGACCAATAAACAGAACTGA | qRT-PCR |
| NDHG qPCR-F |  | GTGGACCATTGGGAATGGGA | qRT-PCR |
| NDHG qPCR-R |  | ACCCCGTACCATGACGTATC | qRT-PCR |
| PSBZ-qPCR-F |  | TGCTTTCCAATTGGCAGTTT | qRT-PCR |
| PSBZ-qPCR-R |  | GTTACTCGACCAACCATCAGG | qRT-PCR |
| PP2AA3-qPCR-F |  | CATGCAATGGTTACAAGACAAGGTT | qRT-PCR |
| PP2AA3-qPCR-R |  | CGAGAAGCGATACTGCACGAA | qRT-PCR |

**Table S3. PCR and sequencing primers used in the RNA editing analysis.**

| No. | Editing site | Forward  primer | Primer sequence (F)  5' - 3' | Reverse  primer | Primer sequence (R)  5' - 3' | Sequencing  primer name | Sequencing primer  sequence 5' - 3' |
| --- | --- | --- | --- | --- | --- | --- | --- |
| 1 | accD-794 | accD-F | GTGGATTCAATGCGACAAT | accD-R | ATATGCAAGCAAGGGAGG | accD-956SR | CCGTTTAGTTGACCTGTACCTGTTT |
| 2 | accD-1568 |  |  |  |  | accD-1285SL | GCATTTGCGGGTAAAAGAGT |
| 3 | atpF-92 | atpF-92F | CCGATTCTTTCGTTTACTTG | atpF-92R | AGGGTTCCTATAGCTCCTTG | atpF-92F | CCGATTCTTTCGTTTACTTG |
| 4 | clpP-559 | clpP-559F | ATGATCCATCAACCCGCTAG | clpP-559R | TATTGAACCGCTACAAGATC | clpP-397SL | TATGAGGCACAAACGGGAGA |
| 5 | matK-640 | matK-640F | CGTTACCGGGTAAAAGATGC | matK-640R | AGCGGCGTATCCTTTGTTGC | matK-813SL | TTTTCCATAGAATACAATTCGCTCA |
| 6 | ndhF-290 | ndhF-290F | AAAACCTTCGCCGCATGTGG | ndhF-290R | GCATTCGCTGCAATAGGTCG | ndhF-290R | GCATTCGCTGCAATAGGTCG |
| 7 | ndhG-50 | ndhG-50F | ATAATGGATTTGCCTGGACC | ndhG-50R | CTTATTAAATCTTGCTCTAGAATCTGGTT | ndhG-312SR | ACAAACCAACGAAGTAATCCCA |
| 8 | petL-5 | petL-5F | AGGGAAGTACTTTAAGAATC | petL-5R | ATTAGACCTAAGACGATTCC | petL-175SR | ACACGGTAAGGAACTATCGAACA |
| 9 | psbE-214 | psbE-214F | ACAGGAGAACGTTCTTTTGC | psbE-214R | CGTTGGATGAACTGCATTGC | psbE-214F | ACAGGAGAACGTTCTTTTGC |
| 10 | psbF-77 |  |  |  |  |  |  |
| 11 | psbZ-50 | psbZ-50F | AGAACATAGCCCTATGAGTT  AATACGA | psbZ-50R | GATAAGAGAATTAAGGATA  CCCACCA | psbZ-SL | GCTATGAGTTAATACGATCCCTA |
| 12 | rpl23-89 | rpl23-89F | AATTCCTACTGGATGCACGC | rpl23-89R | AAGAGGTGGAATAGAATAACCCG | rpl23-89R | AAGAGGTGGAATAGAATAACCCG |
| 13 | rpoA-200 | rpoA-200F | CGGACACTACAGTGGAAGTG | rpoA-200R | ATGAATACAGCATCGATAGG | rpoA-384SR | TTCCACAGCGGGCGGTAA |
| 14 | rpoB-338 | rpoB-338F | TATCGGTTTATTGATCAGGG | rpoB-338R | GCAGCTGCTAACACATCTCG | rpoB-193SL | AAAGAACGAGATGCTGTCTATGAA |
| 15 | rpoB-551 |  |  |  |  |  |  |
| 16 | rpoB-2432 | rpoB-2432F | AACACCTCAAGTGGCGAAAG | rpoB-2432R | GTCCTACATTCATGCGTGAG | rpoB-2244SL | GGCGAAAGAATCCTCCTATGC |
| 17 | rpoC1-488 | rpoC1-488F | TTTTCTTTTGCTAGGCCCATAA | rpoC1-488R | TTCGCAAATCTAAATCGGCT | rpoC1-593SR | GCACCCGCCCCAGTAGAA |
| 18 | rps12-i-58 | rps12-intF | CAAGACAGCCAATCCGAAAC | rps12-intR | CTTGTACAATTCACATTCTTTGGC | rps12-int-SR | TTTACCCTGTTAGTCCGTTCTTTTC |
| 19 | rps14-80 | rps14-80F | TTGATTTATAGGGAGAAGAAGAG | rps14-149R | TACCAGCTTGATCTTGTTGC | rps14-266SR | GCCTGAACCATTTCCCGAAG |
| 20 | rps14-149 |  |  |  |  |  |  |
| 21 | ndhB-149 | ndhB-1F | GCCTTTCATTTGCTTCTCTT | ndhB-1R | TCCTTCGTATACGTCAGGA | ndhB-346SR | CGGATAGAGGAATACAGAGAGTTGA |
| 22 | ndhB-467 |  |  |  |  | ndhB-271SL | TTCCAAACGAACAATTTCAACG |
| 23 | ndhB-586 |  |  |  |  |  |  |
| 24 | ndhB-746 |  |  |  |  |  |  |
| 25 | ndhB-830 | ndhB-2F | CGTATACGAAGGATCTCCCAC | ndhB-2R | CTAGAAGCTAAAAAGGGTATCCT | ndhB-1041SR | CCACCATTTGAGTCTCCAACA |
| 26 | ndhB-836 |  |  |  |  |  |  |
| 27 | ndhB-872 |  |  |  |  |  |  |
| 28 | ndhB-1255 |  |  |  |  | ndhB-1018SL | ATTGTTGGAGACTCAAATGGTGG |
| 29 | ndhB-1481 |  |  |  |  |  |  |
| 30 | ndhD-2 | ndhD-F | TTGAGTACGCGTTCTTTGGAC | ndhD-R | AATAGCTCCATTAAGTCCAGG | ndhD-252SR | TCCATCTATTCCCATTCTCCAGTA |
| 31 | ndhD-383 |  |  |  |  | ndhD-303SL | TTTAGCGGCTTTTCCAGTTAC |
| 32 | ndhD-674 |  |  |  |  |  |  |
| 33 | ndhD-878 |  |  |  |  |  |  |
| 34 | ndhD-887 |  |  |  |  |  |  |
| 35 | ycf3-43350 | ycf3-2iF | CTCAATACCTTCGGGGATTAC | ycf3-2iR | TGCGGAAGCATTACAGAATT | ycf3-2iR | TGCGGAAGCATTACAGAATT |
